# Pro-Survival Role for Parkinson's Associated Gene DJ-1 Revealed in Trophically Impaired Dopaminergic Neurons

**DOI:** 10.1371/journal.pbio.1000349

**Published:** 2010-04-06

**Authors:** Liviu Aron, Pontus Klein, Thu-Trang Pham, Edgar R. Kramer, Wolfgang Wurst, Rüdiger Klein

**Affiliations:** 1 Department of Molecular Neurobiology, Max Planck Institute of Neurobiology, Martinsried, Germany; 2 Helmholtz Center Munich, Technical University of Munich, National Center for Dementia Research, Neuherberg, Germany; 3 Center for Molecular Neurobiology, Hamburg, Germany; Cardiff University, United Kingdom

## Abstract

The mechanisms underlying the selective death of substantia nigra (SN) neurons in Parkinson disease (PD) remain elusive. While inactivation of DJ-1, an oxidative stress suppressor, causes PD, animal models lacking DJ-1 show no overt dopaminergic (DA) neuron degeneration in the SN. Here, we show that aging mice lacking DJ-1 and the GDNF-receptor Ret in the DA system display an accelerated loss of SN cell bodies, but not axons, compared to mice that only lack Ret signaling. The survival requirement for DJ-1 is specific for the GIRK2-positive subpopulation in the SN which projects exclusively to the striatum and is more vulnerable in PD. Using *Drosophila* genetics, we show that constitutively active Ret and associated Ras/ERK, but not PI3K/Akt, signaling components interact genetically with DJ-1. Double loss-of-function experiments indicate that DJ-1 interacts with ERK signaling to control eye and wing development. Our study uncovers a conserved interaction between DJ-1 and Ret-mediated signaling and a novel cell survival role for DJ-1 in the mouse. A better understanding of the molecular connections between trophic signaling, cellular stress and aging could uncover new targets for drug development in PD.

## Abstract 2

A mouse genetic study reveals a novel cell-survival role for the Parkinson's disease-associated gene DJ-1 in dopaminergic neurons that have reduced support from endogenous survival factors.

## Author Summary

The major pathological event in Parkinson disease is the loss of dopaminergic neurons in a midbrain structure, the substantia nigra. The study of familial Parkinson disease has uncovered several disease-associated genes, including DJ-1. Subsequent studies have suggested that the DJ-1 protein is a suppressor of oxidative stress that might modify signaling pathways that regulate cell survival. However, because animal models lacking DJ-1 function do not show dopaminergic neurodegeneration, the function(s) of DJ-1 in vivo remain unclear. Using mouse genetics, we found that DJ-1 is required for survival of neurons of the substantia nigra only in aging conditions and only in neurons that are partially impaired in receiving trophic signals. Aging mice that lack DJ-1 and Ret, a receptor for a neuronal survival factor, lose more dopaminergic neurons in the substantia nigra as compared with aging mice that lack only Ret. Using the fruit fly *Drosophila*, we determined that DJ-1 interacts with constitutively active Ret and with its associated downstream signaling pathways. Therefore, understanding the molecular connections between trophic signaling, cellular stress and aging could facilitate the identification of new targets for drug development in Parkinson Disease.

## Introduction

Specific and progressive loss of substantia nigra (SN) neurons is the central pathogenic event in Parkinson disease (PD), the most common movement neurodegenerative disorder, characterized by tremor, rigidity, and bradykinesia. A second pathological feature of PD is the presence of aggregated alpha-synuclein (Lewy Bodies) in the remaining SN neurons. In most PD patients the degree of dopaminergic axon degeneration in the SN target area, the striatum, exceeds that of SN cell body loss, suggesting a “dying back” model, whereby the axonal compartment is the first target of degenerative insults [1]. A major advance in PD research was the discovery of familial PD-associated genes and the characterization of their biochemical mechanisms [2],[3]. So far, transgenic mouse models that reproduce the genetic defects found in familial PD showed limited power in reproducing disease pathology, and most of them fail to exhibit degeneration of SN neurons [4] (see also [5]). This together with the fact that familial PD accounts for less than 10% of all PD cases (the rest being sporadic) suggests that multiple hits, including environmental factors, underlie selective neuronal death [6].

Access to neurotrophic factors is critical for maintenance of the nigrostriatal system in mice, and novel neurotrophic factors for SN neurons have recently been described [7]–[9]. We recently showed that genetic ablation of the receptor tyrosine kinase (RTK) Ret, the signaling receptor of glial cell line-derived neurotrophic factor (GDNF), led to adult-onset progressive and specific degeneration of the nigrostriatal system [10]. Consistent with a “dying back” model, Ret function was found to be important for striatal DA fiber maintenance, while its role in cell body survival was relatively moderate. Removal of GDNF in the adult brain led to more pronounced degeneration [8], suggesting that SN neurons in Ret mutant mice still have access to trophic support via Ret-independent pathways [11].

The PD-associated gene DJ-1 [12] encodes a small, dimeric, single domain protein that is thought to respond to oxidative stress and to protect neurons from environmental toxins [2],[13]–[16]. However, the molecular mechanisms underlying DJ-1 function are unclear. DJ-1 is localized to cytoplasm, nucleus, and mitochondria, and in each of these subcellular localizations DJ-1 may be neuroprotective [3],[17]. DJ-1 is an oncogene and was shown to synergize with the Ras/MAPK pathway in controlling cellular transformation [18]. It was suggested to negatively regulate the tumor suppressor PTEN, the major negative regulator of the phosphatidylinositol (PI)-3 kinase pathway [19]. DJ-1 ablation in mice alone did not affect the survival of SN neurons [20]–[25] but rendered SN neurons more sensitive towards the toxin MPTP [22]. A small (7%) population of ventral tegmental area (VTA) neurons requires DJ-1 during development for tyrosine hydroxylase (TH) expression [25]. Hence, it is currently not understood why loss of DJ-1 in humans causes specific loss of SN neurons.

Based on the capacity of DJ-1 to interact with pathways implicated in RTK signaling (PI3K/Akt and Ras/MAPK) [18],[19],[26], we investigated a possible cooperation between Ret and DJ-1 in regulating SN neuron survival in vivo. To this end, we generated double mutant mice lacking expression of Ret in midbrain dopaminergic neurons and DJ-1 in all cells of the body (*DAT-Cre;Ret^lx/lx^;DJ-1* ^−*/*−^ mice, in short *DAT-Ret;DJ-1* mice). Here we show that *DAT-Ret;DJ-1* mice have significantly fewer nigral DA neurons than either single mutant, indicating that under conditions of trophic impairment, DJ-1 promotes survival of aging DA neurons. Remarkably, the loss is specific to GIRK-2 positive SN neurons, which project exclusively to the striatum and are more vulnerable in PD. Moreover, DJ-1 does not appear to promote target innervation, suggesting that DJ-1 acts at the level of the DA cell body, not in the axon. To understand how Ret-mediated trophic support relates molecularly to DJ-1, we used *Drosophila* whose genome contains two genes, termed *DJ-1A* and *DJ-1B*, which share significant homology with human *DJ-1*. *Drosophila DJ-1* mutants have been shown to be sensitive to environmental toxins associated with PD [15] and to genetically interact with the PI3K/PTEN/Akt signaling pathway [19],[26]. Our genetic interaction studies in the eye system revealed that *DJ-1A/B* interact genetically with constitutively active Ret and associated Ras/MAPK, but not PI3K/Akt signaling. Paralleling our mouse results, we found that combined deletion of ERK and DJ-1 in *Drosophila* enhanced the developmental defects during eye and wing development caused by ERK deletion, providing evidence for an important role for the interaction between DJ-1 and RTK-related signaling during evolution.

## Results

### Marked Loss of SN Neurons in Mice Lacking Ret and DJ-1

**Figure 1:**
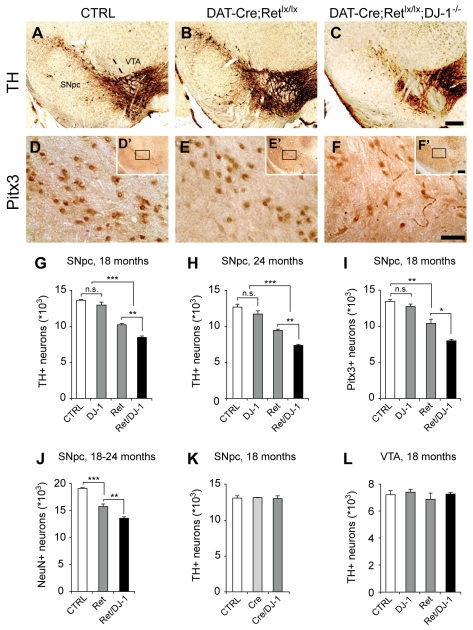
Progressive loss of nigral DA neurons in *DAT-Ret;DJ-1* mice. (A–F) Photomicrographs of 18-mo-old control (wild-type, heterozygous, and *Ret^lx/lx^* littermates; A,D), *DAT-Ret* (B,E), and *DAT-Ret;DJ-1* (C,F) double mutant coronal brain sections showing DA neurons in the SNpc and the VTA stained for the dopaminergic markers TH (A–C) and Pitx3 (D–F). The images in panels D–F are higher magnification views of the stippled area in the insets of each panel. Scale bars: (c, f′) 250 µm, (f) 50 µm. (G–L) Stereological quantifications of TH- and Pitx3-immunoreactive DA neurons in the SN (G–I,K), and NeuN-immunoreactive (DA and non-DA) neurons in the SN (J), and TH-immunoreactive DA neurons in the VTA (L) of the indicated ages and genotypes (*n* = 5–7 mice per genotype; n.s., not significant;* *p*<0.05, ** *p*<0.01, *** *p*<0.001, *t* test). The control groups include wild-type, heterozygous, and *Ret^lx/lx^* littermates.

We have previously shown that the Ret protein co-localizes with the dopaminergic marker TH [10]. Similarly, DJ-1 is expressed in SN and VTA neurons [27]. We determined by Western blotting that the expression of DJ-1 was not modified in the midbrain and striatum of aged *DAT-Ret* mice and vice versa (Figure S1). In cultured SH-SY5Y neuroblastoma cells, endogenous Ret expression was not modified when DJ-1 expression was downregulated by RNAi, nor were the levels of endogenous DJ-1 changed when these cells were stimulated with GDNF (Figure S1); thus, Ret and DJ-1 protein levels appear to be regulated by separate mechanisms. *DAT-Ret;DJ-1* double mutant mice are viable and fertile. To detect morphological alterations in the nigrostriatal system, brain tissue sections of mutant and control mice were immunostained for TH and subjected to stereological quantification. In 3-mo-old *DAT-Ret;DJ-1* double mutant mice, the numbers of TH-positive SN neurons were unchanged compared to age-matched controls (13,690±428 in control and 13,709±248 in *DAT-Ret;DJ-1* mice, *n* = 3 mice/group, *p* = 0.95, student's *t* test) indicating that the nigrostriatal system developed normally in these mutants. When mutant mice were aged, however, the numbers of TH-positive SN neurons decreased significantly compared to age-matched controls (Figure 1A–C,G,H). In *DAT-Ret;DJ-1* double mutant mice, the reduction was more pronounced (37% at 18 mo, 41% at 24 mo) than in *DAT-Ret* single mutant mice (24% at 18 mo, 25% at 24 mo). The difference between *DAT-Ret;DJ-1* double and *DAT-Ret* single mutant mice was statistically significant (*p*<0.01, *t* test) and was not additive, since DJ-1 single mutants had normal numbers of TH-positive neurons (Figure 1G,H). Anti-Pitx3 immunostaining was used as an independent marker and revealed a similar reduction in SN neurons in *DAT-Ret;DJ-1* double and *DAT-Ret* single mutant mice (Figure 1D–F,I). Because approximately one third of the neurons in the SN are non-dopaminergic, we also used the pan-neuronal marker NeuN to label all neurons in the SN and found that aged *DAT-Ret;DJ-1* double mutant mice had significantly fewer neurons in the SN relative to *DAT-Ret* or control mice (Figure 1J). Since the analysis of TH-, Pitx3-, or NeuN-immunolabeled neurons yielded similar numbers of missing neurons in *DAT-Ret;DJ-1* mice, we conclude that combined deletion of Ret and DJ-1 causes enhanced degeneration of SN neurons, relative to deletion of Ret alone. As we had previously shown for *DAT-Ret* single mutants [10], the observed defects were region specific: The nearby VTA region was not affected in *DAT-Ret;DJ-1* double mutants (Figure 1L). The previously observed small (7%) decrease in TH-positive VTA neurons in DJ-1^−/−^ mice [25] was not seen in this analysis, possibly because of a small shift in genetic background due to the presence of the *Ret^lx^* allele. Finally, we excluded that the *DAT-Cre* transgene and the mutant *DJ-1* allele somehow genetically interacted by comparing the numbers of TH-positive neurons in *DAT-Cre;DJ-1* ^−*/*−^ compound mice to *DAT-Cre* transgenics and littermate controls (wild-type and DJ-1^+/−^ mice; Figure 1K). Together these results indicate a requirement for endogenous DJ-1 in maintaining SN neurons, when they are impaired in receiving Ret-mediated trophic signals.

### Ret and DJ-1 Maintain SN Neurons that Express GIRK2

**Figure 2:**
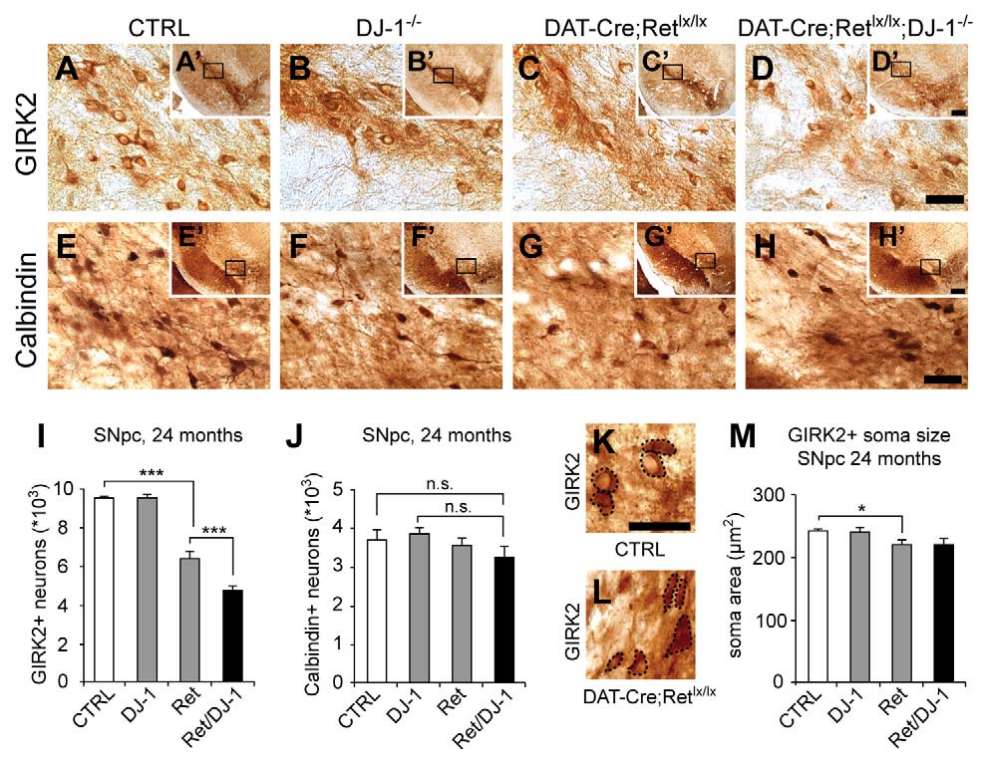
Ret and DJ-1 are required for maintenance of GIRK-2-, but not calbindin-positive neurons. (A–H) Photomicrographs of 24-mo-old control (A,E), *DJ-1* ^−*/*−^ (B,F), *DAT-Ret* single (C,G), and *DAT-Ret;DJ-1* (D,H) double mutant coronal brain sections stained for GIRK2 (A–D) and calbindin (E–H). All images are higher magnification views of the stippled area in the insets of each panel. Scale bars: (d,h) 50 µm, (d′,h′) 250 µm. (I,J) Stereological quantification of GIRK-2 (I) and calbindin-immunoreactive neurons (J) in the SN of 24-mo-old mice of the indicated genotypes (*n* = 5 mice per genotype; n.s., not significant; * *p*<0.05, ** *p*<0.01, *** *p*<0.001, *t* test). (K–M) Photomicrographs of 24-mo-old control (K) and *DAT-Ret;DJ-1* (L) double mutant coronal brain sections stained for GIRK2. Cell somas are indicated by stippled lines. Quantification of soma sizes (M). While remaining *DAT-Ret* SN neurons have a reduced average soma size, no further reduction is seen in *DAT-Ret;DJ-1* double mutant mice (*n* = 5 mice per genotype; * *p*<0.05, *t* test). Scale bar: (K) 50 µm.

Next we asked which SN subpopulation was affected by DJ-1: A9 neurons located in the ventral tier of the SN and projecting to the dorsal striatum are preferentially lost in PD [28]. They express the G-protein gated, inwardly rectifying potassium channel GIRK2 [29]. A9 neurons located in the dorsal tier of the SN and A10 neurons of the VTA project to different areas including limbic and neocortical regions. They express the calcium-binding protein Calbindin [30]. In 24-mo-old mice, removal of DJ-1 had no effect on the number of GIRK2-positive neurons as compared to littermate controls (Figure 2A,B,I). In contrast, removal of Ret alone caused a partial reduction of GIRK2-positive neurons (33% loss) and combined removal of Ret and DJ-1 had the strongest effect (51% loss; *p*<0.001 *DAT-Ret;DJ-1* double versus CTRL; *p*<0.01 *DAT-Ret;DJ-1* double versus *DAT-Ret* single mutants, *t* test; Figure 2C,D,I). Interestingly, the Calbindin-positive subpopulation in the SN was unaffected in all groups (Figure 2E–H,J). Our stereological quantifications revealed that approximately 9,600 SN neurons express GIRK2, while the remaining 3,700 neurons express Calbindin (Figure 2). If all 5,500 TH-positive neurons that were lost in *DAT-Ret;DJ-1* mice were also GIRK2-positive, we would have expected a 57% loss of GIRK2 neurons (5,500 out of 9,600) and no loss of Calbindin-positive neurons. If, however, both populations had been equally vulnerable, we would have expected a 41% loss in both populations. The observed 51% loss in the GIRK2 subpopulation and no statistically significant loss of Calbindin-positive neurons suggest a much higher vulnerability of the GIRK2 subpopulation in *DAT-Ret;DJ-1* and *DAT-Ret* mice. The quantification of soma sizes of surviving GIRK2-positive neurons revealed a small but significant reduction (9%) of the mean soma size in *DAT-Ret* single mutants compared to control littermates; this effect was not further enhanced in *DAT-Ret;DJ-1* double mutants (Figure 2K–M).

### No Requirement for DJ-1 in Maintaining Dopaminergic Nerve Terminals in the Striatum

**Figure 3:**
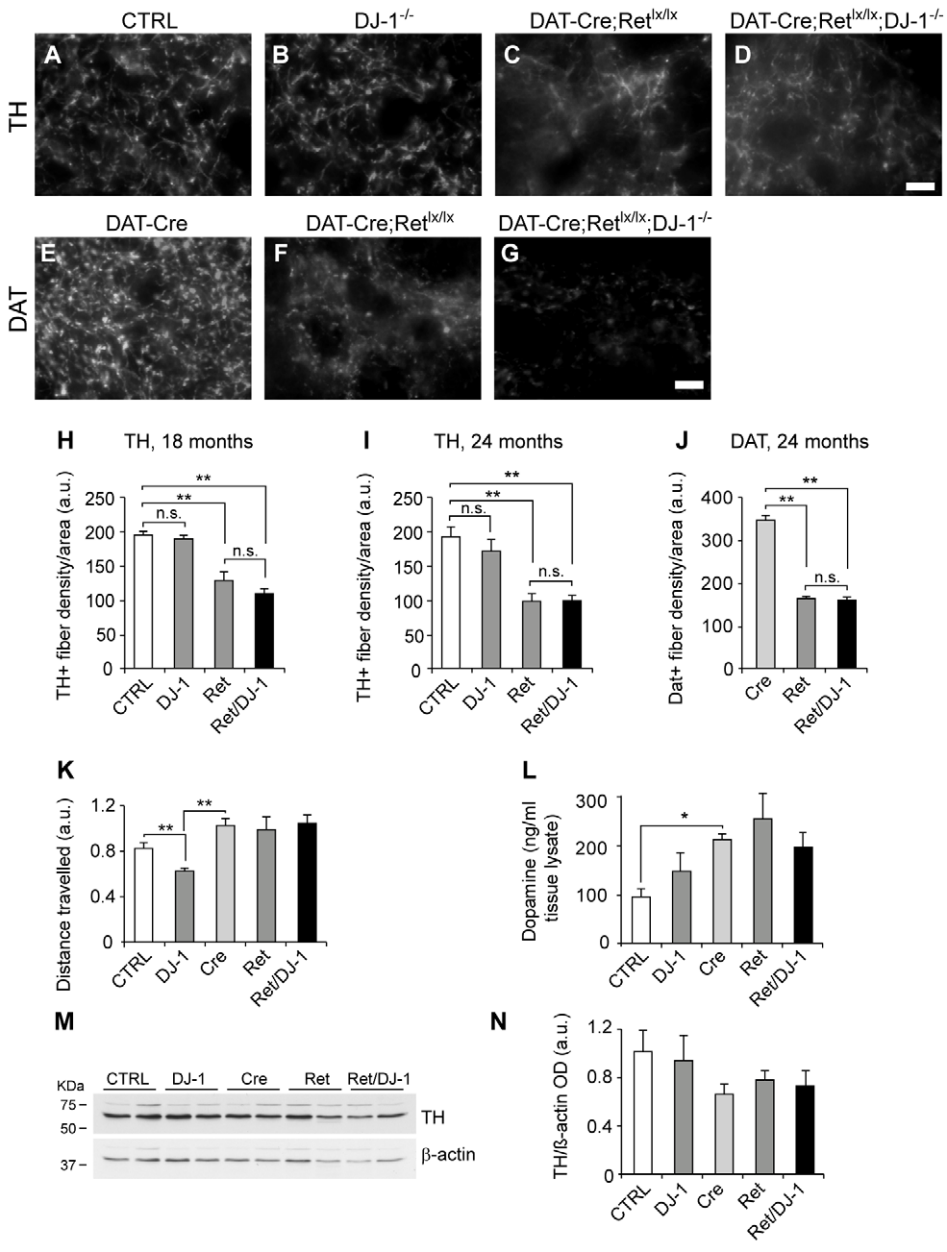
DJ-1 is not required for maintenance of striatal dopaminergic fibers. (A–G) Photomicrographs of immunofluorescence labeling for TH (A–D) and DAT (E–G) of striatal sections of 24-mo-old control (A,E), DJ-1 ^−/−^ (B), DAT-Ret single (C,F), and *DAT-Ret;DJ-1* (D,G) double mutant mice. Scale bars: (d,g) 10 µm. (H,I) Quantification of TH-fiber density in the dorsal striatum of 18-mo- (H) and 24-mo-old mice (I) of the indicated genotypes (*n* = 5–7 mice per genotype; n.s., not significant; ** *p*<0.01, *** *p*<0.001, *t* test). (J) Quantification of DAT-fiber density in the dorsal striatum of 24-mo-old mice of the indicated genotypes (*n* = 5–7 mice per genotype; *** *p*<0.001, *t* test). (K) Open field behavioral assessment of 18-mo-old animals revealed that *DJ-1* ^−*/*−^ mice were significantly impaired in their motor abilities compared to controls (*n* = 11, ** *p*<0.01, *t* test). The absence of one DAT copy in DAT-Cre mice renders them slightly hyperactive (*n* = 12, ** *p*<0.01, *t* test). *DAT-Ret^lx/lx^* and *DAT-Ret^lx/lx^;DJ-1* ^−*/*−^ mice are not further impaired compared to DAT-Cre mice (*n* = 7–16, *p* = n.s.). (L) Measurements of total dopamine levels in the striatum of 18-mo-old mice using HPLC detection. Levels of total dopamine were not significantly increased in *DAT-Ret* and *DAT-Ret;DJ-1* mice relative to control mice carrying the DAT-Cre transgene (Cre) (*n* = 4–8 mice per group). All mice lacking one copy of the DAT had increased levels of dopamine relative to controls (*n* = 4–8 mice, * *p*<0.05, *t* test). (M,N) Western blot analysis of TH expression in the striatum of 24-mo-old mice of indicated genotypes (*n* = 2 mice). β-actin was used as loading control. Quantifications of several blots did not reveal significant differences between the different mouse mutants (N; *n* = 5–7 mice per group).

We next evaluated the possibility that Ret and DJ-1 cooperate in maintaining target innervation of nigral DA neurons. The quantification of TH-positive fiber density confirmed a marked decrease in the dorsal striatum of 18- and 24-mo-old *DAT-Ret* single mutants compared to age-matched controls (Figure 3A,C,H,I; see also [10]). In contrast, no significant reductions of TH-positive fibers were observed in DJ-1 single mutant mice (Figure 3B,H,I). Interestingly, *DAT-Ret;DJ-1* double mutants displayed reductions of TH-positive fibers that were not significantly different from *DAT-Ret* single mutants (46% at 18 mo and 52% at 24 mo, Figure 3D,H,I). Similar results were obtained when the dopamine transporter (DAT) protein was used as an independent marker for DA terminals (54% reduction in both mutant lines; Figure 3E–G,J). In this case the DAT-Cre knock-in mice were used as controls, since they have reduced levels of DAT protein (unpublished data) due to the loss of one functional copy of the DAT gene. These results indicate that DJ-1 is not required for maintaining target innervation in DA neurons that are partially impaired in receiving trophic support. To evaluate the motor performance of aged mutant mice, we followed their horizontal activity in an open field arena. Consistent with previous observations [21], *DJ-1* null mice were found to be hypoactive, despite having normal numbers of SN neurons and normal target innervation (Figure 3K). Mice carrying the DAT-Cre transgene inserted into the 5′ UTR of the DAT gene were slightly hyperactive (Figure 3K), in agreement with previous reports [31]. Removal of Ret or Ret and DJ-1 function did not further modify motor behavior compared to DAT-Cre control mice (Figure 3K). We then measured the levels of total striatal dopamine in these mutants and found a significant increase in dopamine levels in mice carrying the DAT-Cre transgene, while removal of Ret or Ret and DJ-1 did not further modify these levels compared to DAT-Cre control mice (Figure 3L). The TH enzyme is a critical regulator of dopamine production in DA neurons, and our analysis of TH levels in the different aging mutants revealed no significant differences in TH levels (Figure 3M,N). Taken together, these results suggest the existence of compensatory mechanisms that maintain dopaminergic homeostasis in *DAT-Ret* and *DAT-Ret;DJ-1* mice, despite the occurrence of partial neurodegeneration in the SN and the striatum.

### No Enhanced Neuroinflammation in the Striatum of *DAT-Ret;DJ-1* Mice

**Figure 4:**
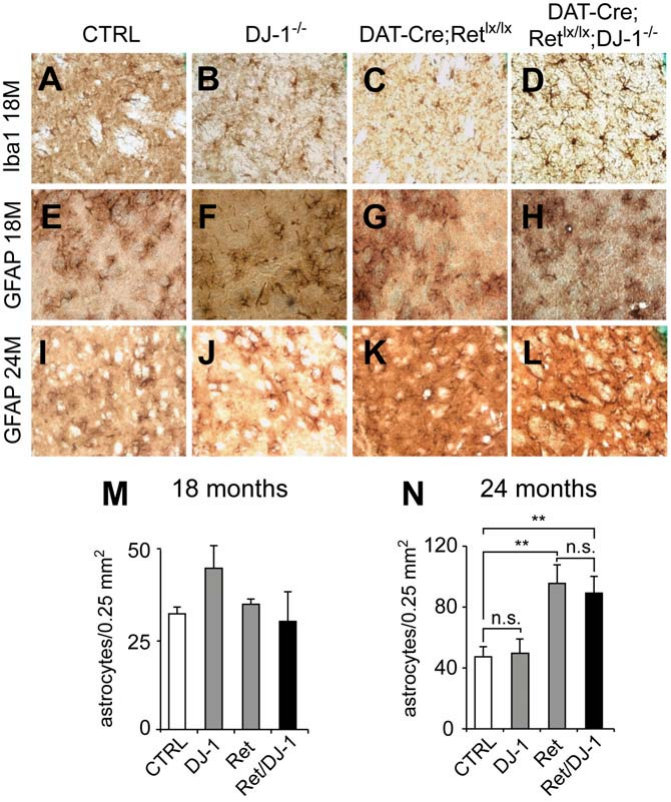
No enhanced neuroinflammation in the striatum of aged *DAT-Ret;DJ-1* mice. (A–L) Photomicrographs of 18-mo-old (A–H) and 24-mo-old (I–L) control (A,E,I), *DJ-1* ^−*/*−^ (B,F,J), *DAT-Ret^lx/lx^* (C,G,K), and *DAT-Ret^lx/lx^;DJ-1* ^−*/*−^ (D,H L) mouse dorsal striatal sections stained for the microglial marker Iba-1 (A–D) and the astrocytic marker GFAP (E–L). While the density of microglia and astrocytes is similar at 18 mo in all groups, there is an increased astrocyte density in *DAT-Ret^lx/lx^* (K) and *DAT-Ret^lx/lx^;DJ-1* ^−*/*−^ (L) at 24 mo compared to control (I) and *DJ-1* ^−*/*−^ mice (J). (M,N) Quantifications of astrocyte densities at 18 and 24 mo. (M) At 18 mo, there were no significant differences between control, *DJ-1* ^−*/*−^,*DAT-Ret^lx/lx^*, and *DAT-Ret^lx/lx^;DJ-1* ^−*/*−^ mice (*n* = 4–5 animals/group). (N) At 24 mo, astrocyte densities were increased in both *DAT-Ret^lx/lx^* and *DAT-Ret^lx/lx^;DJ-1* ^−*/*−^ to a similar extent as compared to control and *DJ-1* ^−*/*−^ mice (*n* = 4–5 animals/group; ** *p*<0.01, *t* test).

Dopaminergic-specific deletion of Ret leads to enhanced astrogliosis, but not microglial recruitment in the striatum of 24-mo-old mice (Figure 4 and [10]). Using the microglial marker Ionized binding calcium adapter molecule (Iba-1) and the astrocytic marker glial fibrillary acidic protein (GFAP), we evaluated the occurrence of neuroinflammatory processes in the striatum of aged *DAT-Ret;DJ-1* mice and corresponding controls. We found no enhanced recruitment of Iba-1-positive microglial cells in *DAT-Ret;DJ-1* or *DAT-Ret* mice compared to controls (Figure 4A–D). The recruitment of reactive astrocytes in the *DAT-Ret* striatum was found to be significantly elevated after 24 mo, while additional removal of *DJ-1* did not enhance this process (Figure 4E–N). These observations correlate with the above-mentioned histological, behavioral, and physiological measurements and suggest that removal of Ret and DJ-1 function does not exacerbate the structural and functional defects in SN axon terminals caused by Ret deprivation.

### Genetic Interaction of DJ-1A/B and dRet in *Drosophila* Eye Development

**Figure 5:**
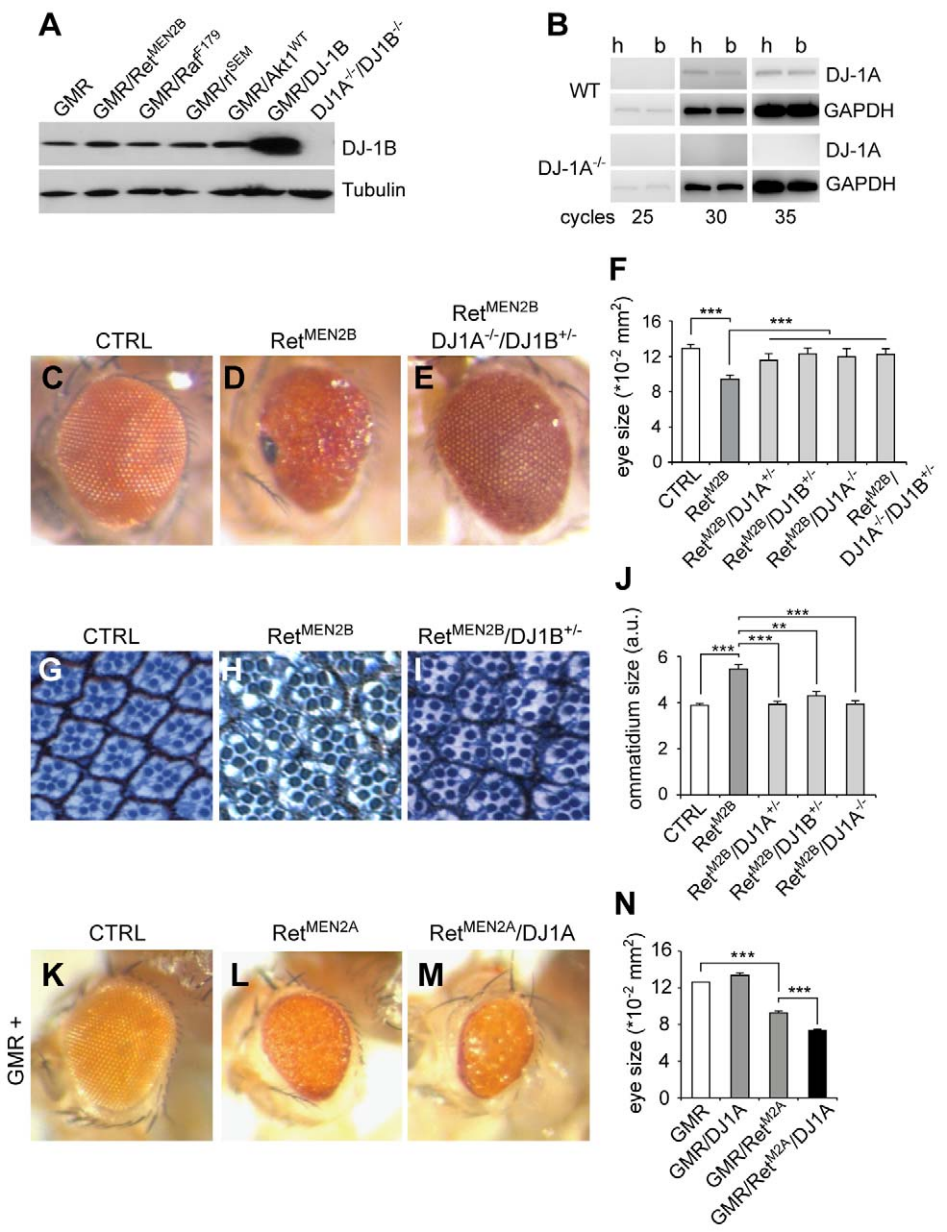
Genetic interaction between dRet and DJ-1A/B in the *Drosophila* eye. (A) Expression of endogenous DJ-1B protein in P1–P5 fly heads. Western blot analysis showing normal levels of endogenous DJ-1B levels after overexpression of constitutively active Ret, Raf, ERK/rl, or wild-type Akt1 (all expressed by the GMR promoter). DJ-1B overexpression leads to an increase in DJ-1B levels, whereas no DJ-1B was detected in *DJ-1A* ^−*/*−^ *DJ1B* ^−*/*−^ heads. (B) RT-PCR analysis for the DJ-1A transcript in adult fly head (H) and body (B). The relative abundance of DJ-1A transcripts in head and body was similar after different cycles of PCR amplification. DJ-1A mRNA is absent in *DJ-1A* ^−*/*−^ flies and GAPDH was used as control. (C–E) Images of the normal eye of an adult control fly (C), the smaller rougher eye of a fly overexpressing dRet^MEN2B^ controlled by the GMR promoter (D), and the near normal eye of a *GMR- dRet^MEN2B^* fly carrying the indicated loss-of-function alleles of *DJ-1A/B* (E). (F) Quantification of eye sizes in the indicated mutant and control flies (*n* = 17–25 eyes per genotype; *** *p*<0.001, *t* test). (G–I) Photomicrographs of ultrathin eye sections stained with toluidine blue showing the normal size and pattern of individual ommatidia of an adult control fly (G), the larger ommatidia of a *GMR-dRet^MEN2B^* fly (H), and the near normal ommatidia of a *GMR- dRet^MEN2B^;DJ-1B^+/^* ^−^ fly (I). (J) Quantification of ommatidia sizes in the indicated mutant and control flies (*n* = 4 eyes per genotype; ** *p*<0.01, *** *p*<0.001, *t* test). (K–N) Overexpression of Ret^MEN2A^ using the GMR promoter (*GMR/UAS-dRet^MEN2A^*) leads to a moderate rough eye phenotype (L) compared to a control fly (K), which can be further enhanced by DJ-1A co-overexpression (M). *GMR/UAS-dRet^MEN2A^/UAS-DJ1A* eyes are rougher, have a glassy appearance (M), and a reduced size (N) (quantification of *n*>10 eyes per genotype, *** *p*<0.001, *t* test).

To obtain independent evidence for genetic interaction between Ret and DJ-1 and to begin characterizing the underlying intracellular pathways, we used the developing *Drosophila* eye system, which is very sensitive to dosage changes in RTK signaling and downstream components of the PI3K/Akt and Ras/Mapk pathways. While *Drosophila* DJ-1B is ubiquitously expressed, DJ-1A appears to be enriched in certain tissues such as testes [14],[15]. We used a DJ-1 specific antibody [15] and confirmed that DJ-1B is expressed at high levels in the adult head; DJ-1A expression was not detected in WB, but the presence of the DJ-1A transcript was confirmed by RT-PCR (Figure 5B) [14]; moreover, overexpression of constitutively active versions of Ret, Raf, ERK/rolled, or wild-type Akt1 did not modify endogenous DJ-1 levels (Figure 5A). Flies homozygous for *DJ-1A* and/or *DJ-1B* null alleles and flies overexpressing DJ-1A or DJ-1B in the eye (using the photoreceptor neuron-specific driver GMR-Gal4) displayed normal eye development and ultrastructure (unpublished data). *Drosophila* Ret (dRet) is highly homologous to mammalian Ret [32] and exhibits activities associated with human Ret both in tissue culture cells and during *Drosophila* eye development [33],[34]. A function for dRet has so far not been described in *Drosophila*, and in addition, dRet does not bind mammalian GDNF; we therefore utilized previously generated constitutively active forms of dRet (dRet^MEN2A/B^) that interact with the same pathways as WT Ret and were used to screen for novel Ret interactors [34]. Flies carrying the GMR driver fused to dRet^MEN2B^ (*GMR-dRet^MEN2B^*) [34] develop with adult eyes of reduced size and rough morphology. Ommatidia sizes were increased by 35% and individual ommatidia were often fused together, had abnormal polarity, and had poorly patterned interommatidial spaces (Figure 5G,H,J). Despite an increase in ommatidia size, the overall eye size in *GMR-dRet^MEN2B^* flies was decreased by 30% compared to controls (Figure 5C,D,F), as a result of a late (pupal) pro-apoptotic wave induced by excessive proliferation and differentiation defects [34]. To determine whether *DJ-1* is a *dRet* interactor, we crossed *GMR-dRet^MEN2B^* flies with flies carrying *DJ-1A* and/or *DJ-1B* microdeletions [15]. Remarkably, the defects in eye and ommatidia sizes induced by overactive dRet were completely rescued in flies with reduced DJ-1A/B levels (Figure 5E,F,I,J). Similar results were obtained with independent *DJ-1A/B* loss-of-function alleles and the *GMR-dRet^MEN2A^* gain-of-function allele (Figure S2). To test whether overexpression of both Ret and DJ-1 led to a more severe phenotype than the ones induced by active dRet alone, we overexpressed DJ-1A in flies with a moderate Ret-overexpression phenotype (*GMR-Gal4/UAS-dRet^MEN2A^*). The resulting flies displayed an enhanced eye phenotype (Figure 5K–N). Thus, both DJ-1A/B interact genetically with overactive dRet in controlling cell size and differentiation in the developing fly retina.

### No Interaction between PI3K/Akt Signaling and DJ-1A/B

**Figure 6:**
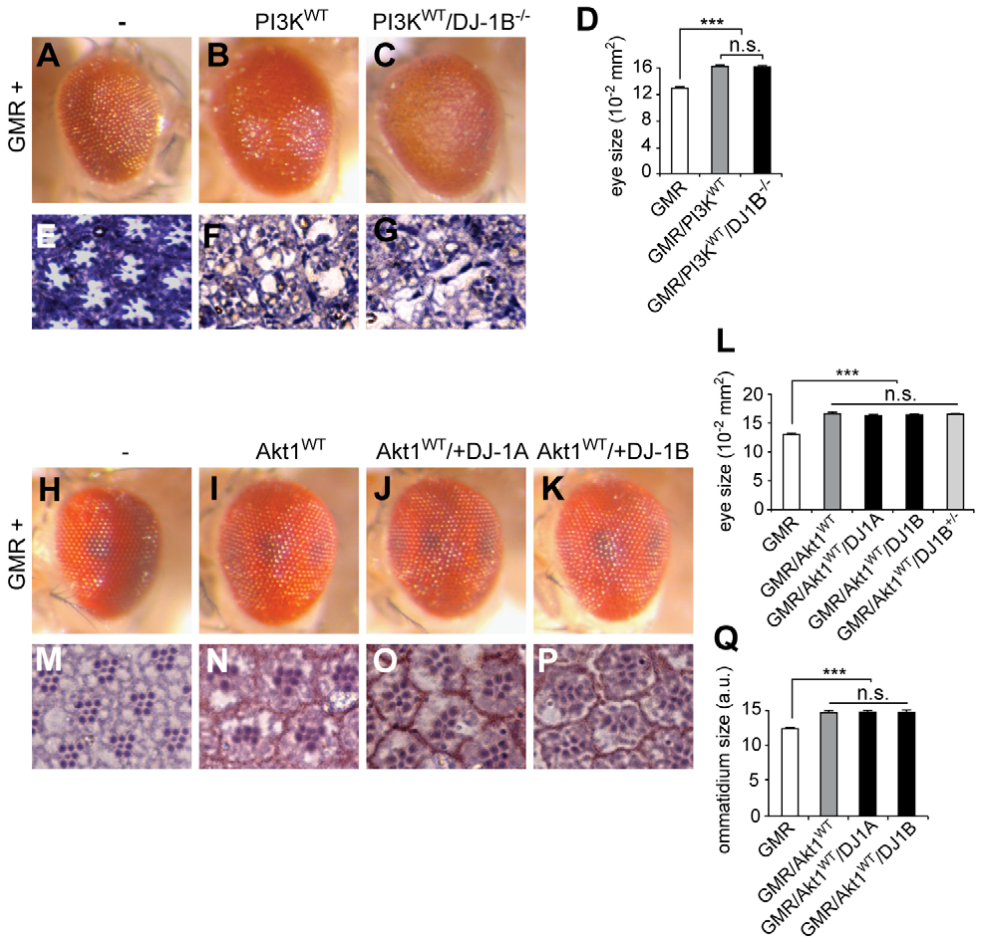
No interaction between PI3K/Akt and DJ-1A/B in the *Drosophila* eye. (A–C) Images of the normal eye of an adult control fly (A), of an increased eye after PI3K^WT^ overexpression (B), and in flies overexpressing of PI3K^WT^ in a *DJ-1B* ^−*/*−^ background (C). Flies were raised at 30°C to enhance the phenotype; similar results were obtained at 25°C (unpublished data). (D) Quantification of eye sizes (*n*>25 eyes per genotype; *** *p*<0.001, *t* test). (E–G) Photomicrographs of ultrathin eye disc sections stained with toluidine blue showing the normal size and pattern of individual ommatidia of an adult control fly (E), the disorganized eyes of a *PI3K^WT^* (F), and a *PI3K^WT^/DJ-1B* ^−*/*−^ fly (G). (H–Q) No enhancement of Akt1^WT^ overexpression phenotype by DJ-1A or DJ-1B co-overexpression or in a heterozygous *DJ-1B* knockout background. The increased eye size compared to controls (H) after Akt1^WT^ overexpression (I) is not further modified after DJ-1A (J) or DJ-1B (K) overexpression nor in a *DJ-1B* heterozygous background (L). Similarly, the increased ommatidial size induced by Akt1^WT^ overexpression (M,N,Q) is not modified by DJ-1A (O,Q) or DJ-1B (P,Q) overexpression nor in a DJ-1B heterozygous background (Q). (L,Q) Quantifications of eye (*n*>25 animals) and ommatidia sizes (*n* = 4 animals analyzed; *** *p*<0.001, *t* test).

To gain insights into the mechanism(s) underlying the genetic interaction between Ret and DJ-1, we investigated the capacity of fly DJ-1A/B to genetically modify pathways that are known to mediate Ret function: PI3K/Akt and Ras/Mapk [33]. Strong overexpression of wild-type PI3K (*GMR/PI3K^WT^* at 30°C) led to a 25% increase in eye size and to a disorganized retina compared to controls (*GMR-Gal4*) (Figure 6A,B,D–F). These phenotypes were not rescued in a *DJ-1B* ^−*/*−^ background (Figure 6C,D,G). To test whether overexpression of DJ-1A/B could enhance the phenotype of increased PI3K/Akt signaling, we used the moderate eye phenotype induced by wild-type Akt1 overexpression (25% increase in eye and 20% increase in ommatidia sizes). The Akt1 overexpression phenotype was not further exacerbated by DJ-1A or DJ-1B overexpression, nor did the resulting eyes become disorganized (Figure 6H–Q). Similar results were obtained in flies expressing a constitutively active version of PI3K (PI3K^CAAX^, [35], Figure S3). Conversely, reduced PI3K activity (using the GMR-driven expression of a PI3K dominant negative version), leading to a moderate reduction in eye size and to loss of photoreceptors, was not further enhanced in a *DJ-1B* null background (unpublished data). Our findings are in apparent contrast to published reports indicating genetic interactions of *Drosophila* DJ-1 with PTEN, an inhibitor of Akt [19], and with mammalian DJ-1 being a modulator of PI3K/Akt signaling in cultured cells [19],[36]. We found that DJ-1A/B overexpression only mildly rescued the effects of PTEN overexpression (reduced eye and ommatidia size) and that a reduction in DJ-1A/B function did not visibly enhance the PTEN overexpression phenotype (Figure S3). Furthermore, our experiments using different cell lines failed to reproduce the previously reported modulation of Akt activation by DJ-1, in conditions of DJ-1 overexpression, RNAi knockdown, or in DJ-1^−/−^ mouse embryonic fibroblasts (MEFs; Figure S4). Thus, our data suggest that DJ-1 does not synergize with PI3K/Akt signaling during eye development, nor does DJ-1 modulate the activation status of Akt under normal conditions. DJ-1 might interact with PTEN only in defined situations (e.g., in oncogenic conditions) but in a PI3K-Akt independent manner (see Discussion).

### DJ-1 Interacts Genetically with the Ras/Mapk Pathway in *Drosophila*

**Figure 7:**
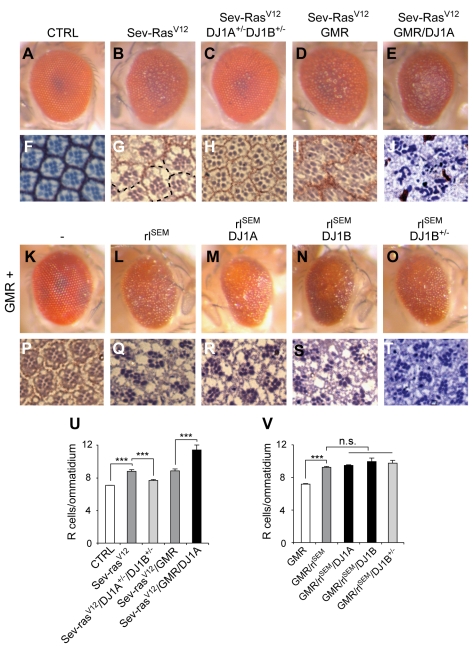
DJ-1A/B interact genetically with Ras signaling in *Drosophila*. (A–J, U) DJ-1A/B interacts genetically with constitutively active Ras in the fly retina. (A–E) Eye images showing a normal control eye (A), the rough eye phenotype induced by activated Ras under the control of the sevenless promoter (*sev-Ras^12V^*, B), and the rescue of the rough eye in a *DJ-1A/DJ-1B* heterozygous knockout background (C). GMR-mediated DJ-1A overexpression further enhances the Ras-induced phenotype (*Sev-Ras^12V^/GMR/DJ1A*, E) as compared to control (*Sev-Ras^V12^/GMR*, D). (F–J) Photomicrographs of ultrathin eye sections showing normal control ommatidia (F), the Sev-Ras^12V^-induced increase in photoreceptors (R cells) and fused ommatidia (G, stippled lines indicate missing separations), and the rescue after reducing DJ-1A/DJ-1B function (H). (J) Further increase of the number of R cells/ommatidium and worsening of retinal ultrastructural defects after DJ-1A overexpression as compared to control (I). (U) Quantification of the numbers of R cells/ommatidium (*n* = 4 animals per group and *n*>150 ommatidia analyzed per animal; ** *p*<0.01 and *** *p*<0.001, *t* test). (K–T, V) No further modulation of constitutively active rolled-induced eye phenotype by DJ-1A or DJ-1B. (K–O) Eye images showing a control eye (K), the rough eye phenotype induced by rl^SEM^ overexpression (L), and the lack of modulation after co-overexpression of DJ-1A (M) or DJ-1B (N) or after decreasing DJ-1B function (O). (P–T) Photomicrographs of ultrathin eye sections showing normal control ommatidia (P), the rl^SEM^-mediated increase in R cell number/ommatidium (Q), and the lack of modulation after co-overexpression of DJ-1A (R), DJ-1B (S), or after decreasing DJ-1B levels (T). (V) Quantification of the numbers of R cells/ommatidium (*n* = 4 animals per group and *n*>150 ommatidia analyzed per animal; *** *p*<0.001, *t* test).

To investigate the interaction of DJ-1 with the Ras/ERK pathway, we used constitutively active versions of Ras and the Mapk ERK/*rolled* (rl), which impair eye development by promoting excessive proliferation and altered cell differentiation [37]. Overexpression of active Ras in R7 photoreceptor neurons with the sevenless promoter (*Sev-Ras^V12^*) led to the induction of multiple R cells/ommatidium and to a rough eye phenotype (Figure 7A,B,F,G,U). This phenotype was rescued by reducing endogenous DJ-1A/B levels (*Sev-Ras^V12^/DJ-1A^+/^* ^−^ */DJ-1B^+/^* ^−^) (Figure 7C,U). *Sev-Ras^V12^* flies displayed on average 8.5 R cells/ommatidium while in *Sev-Ras^V12^/DJ-1A^+/^* ^−^ */DJ-1B^+/^* ^−^ flies only 7.5 R cells/ommatidium were detected (Figure 7H,U). In addition, in *Sev-Ras^V12^* flies, 67% of ommatidia were abnormally fused with their neighbours, compared to only 5% in *Sev-Ras^V12^/DJ-1A^+/^* ^−^ */DJ-1B^+/^* ^−^ flies (Figure 7G,H and unpublished data). Conversely, overexpression of DJ-1A further enhanced the Ras-overexpression phenotype to 11 R cells/ommatidium in *Sev-Ras^V12^/GMR/DJ-1A* retinas (Figure 7D,E,I–U). We next assessed the modulation of constitutively active *rolled* signalling (*GMR/rl^SEM^*) by DJ-1. Overexpression of rl^SEM^ led to supernumerary photoreceptor neurons (9.5 R cells/ommatidium). DJ-1A/B overexpression or partial reduction of DJ-1B levels did not modulate this phenotype (Figure 7K–T,V). Moreover, in cultured cells, increasing or decreasing DJ-1 levels did not modulate the phosphorylation status of ERK1/2 under basal conditions or following stimulation by growth factors (Figure S4; see also [38]). These results suggest that DJ-1A/B function either between Ras and ERK or in parallel to the Ras/ERK pathway to control cell differentiation and proliferation induced by overactive Ras/Mapk signaling.

**Figure 8:**
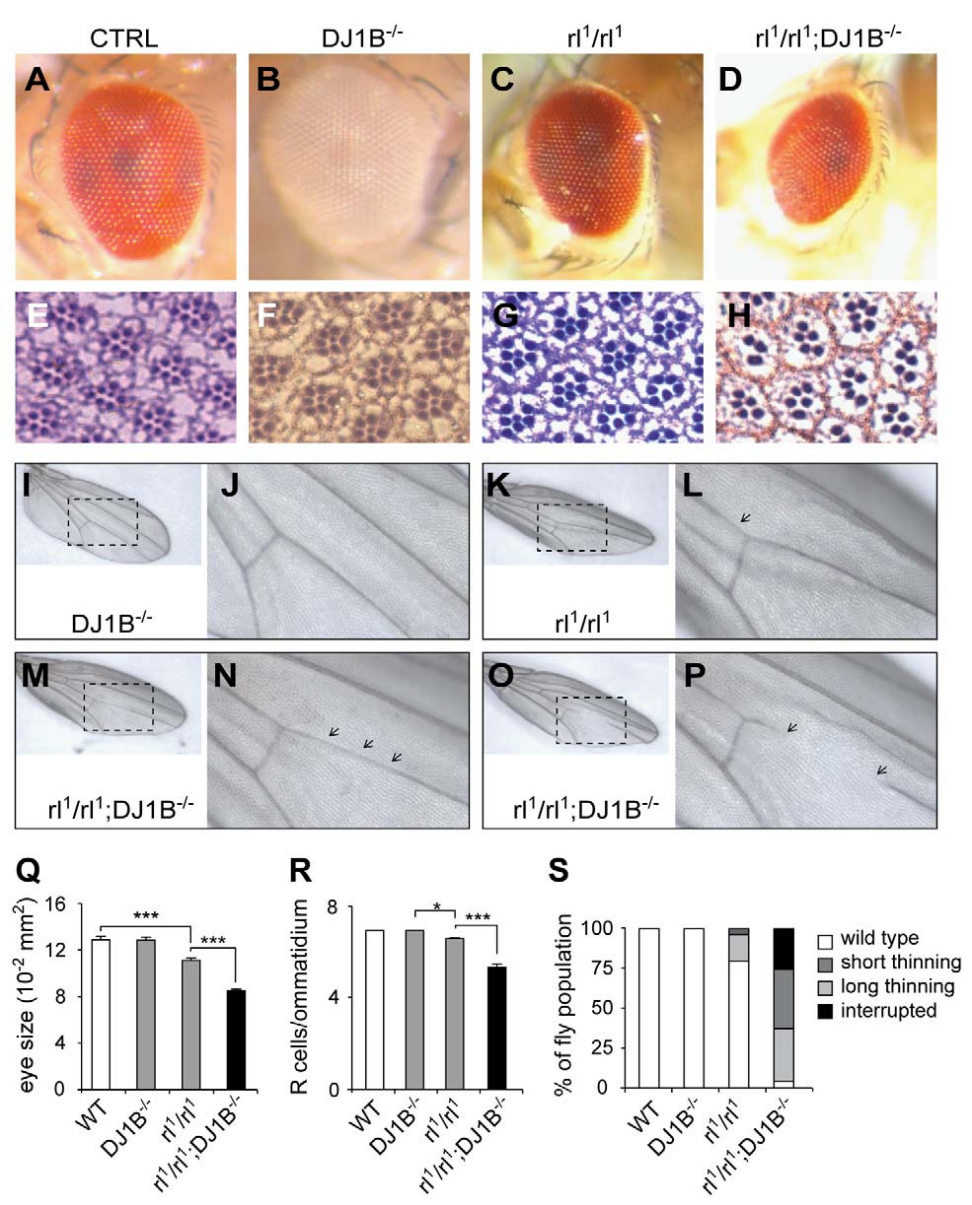
DJ-1B cooperates with endogenous Ras/Mapk signaling to control photoreceptor neuron and wing development. (A–H,Q,R) Interaction between DJ-1B and rolled/ERK controls photoreceptor neuron development. (A–D) Images of normal eyes of control (A) and *DJ-1B* ^−*/*−^ (B) flies, of a smaller *rl^1^/rl^1^* eye (C), and a very small and rough eye in a fly double mutant for rolled and DJ-1B (*rl^1^/rl^1^ DJ1B* ^−*/*−^, D). (Q) Quantification of eye sizes (*n*>50 eyes per group, *** *p*<0.001, *t* test). (E–H) Photomicrographs of ultrathin eye sections showing the normal retina of control (E) and *DJ-1B* ^−*/*−^ flies (F), the mild reduction in R cells/ommatidium in the *rl^1^/rl^1^* retina (G), and the stronger reduction seen in the *rl^1^/rl^1^ DJ1B* ^−*/*−^ retina (H). (R) Quantification of the numbers of R cells/ommatidium (*n* = 4 eyes per animal and *n*>150 ommatidia analyzed per animal, * *p*<0.05 and *** *p*<0.001, *t* test). (I–P,S) Interaction between DJ-1B and rolled/ERK controls wing vein development. (I,K,M,O) Images of full-size wings and (J,L,N,P) magnifications showing the central portion of the L4 vein (arrows). *DJ-1B* ^−*/*−^ flies develop with normal wings (I,J) while in *rl^1^/rl^1^* flies the L4 vein has short stretches of thinner diameter (K,L). In contrast, *rl^1^/rl^1^ DJ1B* ^−*/*−^ flies often develop with the L4 vein having long stretches of thinner diameter (M,N) or with an interrupted L4 vein (O,P). (S) Quantification of the L4 vein phenotype categorized as wild-type, with short stretches of thinner diameter, with long stretches of thinner diameter, or interrupted. The observed frequencies are shown for the different genotypes (*n*>115 wings analyzed per animal).

Our loss-of-function mouse experiments suggest that DJ-1 acts in parallel to Ret-mediated signalling to control dopaminergic neuron survival. The *Drosophila* interactions between DJ-1 and ectopic Ras signalling raise the possibility that DJ-1 acts in parallel to the Ret induced Ras/Erk pathway to control optimal activation of Mapk downstream targets. To test this possibility and to investigate whether DJ-1 interacts with endogenous Erk signalling in *Drosophila*, we performed double loss-of-function experiments. We chose to investigate this interaction in two places where Erk/rolled is known to play a crucial role during development: the development of photoreceptor neurons and wing venation. Flies carrying two hypomorphic *rolled* alleles (rl^1^) displayed a moderate eye phenotype caused by a mild reduction in the number of R cells/ommatidium (6.64; Figure 8A,C,E–G,Q,R). While control and *DJ-1B* ^−*/*−^ flies had a normal appearance and a normal complement of 7 R cells/ommatidium, eyes of *rl^1^/rl^1^;DJ-1B* ^−*/*−^ flies were significantly smaller, rough, and displayed on average only 5.34 R cells/ommatidium (Figure 8A–H,Q,R; *p*<0.05 CTRL versus *rl^1^/rl^1^*; *p*<0.001 *rl^1^/rl^1^* versus *rl^1^/rl^1^ DJ-1B* ^−*/*−^, *t* test). DJ-1B is thus required, as a *rolled* interactor to control photoreceptor neuron development. We then investigated the development of the wing and found that *rl^1^/rl^1^* flies had a very mild defect in wing venation, the vein L4 being sometimes thinner (in about 20% of the animals; Figure 8I–P,S). While in control and DJ-1B^−/−^ flies the L4 vein developed normally, in *rl^1^/rl^1^;DJ-1B* ^−*/*−^ mutants the thinning of the L4 vein was either short (in 33% of animals), long (37% of all cases), or the L4 vein was interrupted (in 25% of animals, Figure 8I–P,S). Such an enhanced phenotype was also seen in flies carrying a combination of rl^1^ and the stronger allele rl^10^ (a deficiency; [37]). DJ-1 is thus required, as a *rolled* interactor, to control the development of the wing. These results uncovered a novel *DJ-1B* ^−*/*−^ phenotype in the unchallenged fly and suggest that DJ-1B cooperates with Ras/Mapk signalling during photoreceptor neuron and wing imaginal disc development.

## Discussion

The mechanisms underlying the selective death of SN neurons in PD are at present unclear. Mutations in DJ-1 cause PD and, although a number of in vitro studies have suggested several functions for DJ-1 in controlling cell survival and stress response, it remained unclear whether DJ-1 plays at all a role in neuronal survival in vivo. To explore the survival function of DJ-1 in aging neurons, we took a genetic approach that in difference to cell culture models allowed us to study the relevance of DJ-1 during the life span of the mouse and within the physiological environment of the brain. Remarkably, we found that DJ-1 becomes critical for survival only under trophic deprivation situations and only during aging. Current clinical trials examine the effects of GDNF (and its relative Neurturin) delivery for PD, although with conflicting results, suggesting that improvements in both technology and biological understanding of GDNF action are required [7],[39]. We have previously shown that GDNF signaling via the Ret RTK is required for maintenance of aging SN neurons [10]. The effect of Ret inactivation on cell survival was relatively moderate compared to its effect on DA axons innervating the striatum. The identification of new interactors for Ret might better define the context in which GDNF delivery is most beneficial and might provide new clues for PD therapy [7]. We show here DJ-1 acts in parallel to Ret-induced pathways to control SN DA neuron survival, and complementary studies in *Drosophila* suggest that the interaction between Ret and DJ-1 involves primarily Ras/ERK signaling. Because Ret and DJ-1 show convergence of their pro-survival activities, we suggest exploring the possibility that GDNF delivery might be most effective in PD patients carrying DJ-1 mutations, i.e. by activation of DJ-1 downstream signaling via Ret activation.

### DJ-1 Promotes Survival of Trophically Impaired Dopaminergic Neurons

Based on these results we propose a model in which DJ-1 primarily promotes the survival of DA neurons that have suffered from an independent hit (trophic insufficiency) and have greatly reduced target innervation. In aged Ret single mutant mice, we have previously shown that the loss of target innervation exceeded cell loss; hence these mice contained a fraction of cells that survived during aging but had strongly reduced target innervation [10]. The present study shows that in aged *DAT-Ret/DJ-1* double mutants, additional cell loss occurs such that the degree of cell loss exactly matches the degree of target innervation loss. The simplest explanation is that additional DJ-1 removal primarily leads to loss of cells that have strongly reduced target innervation (due to Ret signaling loss), while the larger fraction of cells with functional connections to the striatum remains unaffected. Alternatively, DJ-1 removal may affect both cell populations; however, as the projections to the striatum do not decrease further in the double mutant, surviving neurons would have to resprout and innervate the vacated target area to compensate for the expected loss of innervation. Since our previous study [40] showed that Ret signaling controls DA resprouting after toxic lesions, we find the latter explanation less likely.

The fact that removal of DJ-1 in Ret-deficient mice only accelerated the loss of DA cell bodies but not axons suggests that DJ-1 might exert its pro-survival activities in the SN dopaminergic cell body. Neurotrophic factor receptors, such as Ret, are transported from distal sites to the cell body, where they signal to promote survival. Components of the signaling machinery including activated Ras are also transported to the cell body in signaling vesicles [41]. Recent work succeeded in genetically uncoupling the survival requirements for the axon and cell body compartments. Specific molecules primarily regulate maintenance of cell bodies but not axons (including Bax, Bcl2, and JNK), further supporting the notion that different survival mechanisms operate in these two neuronal compartments [42]. Understanding the differential vulnerability of the axonal and cell-body compartments to aging and degenerative insults might improve our understanding of neurodegeneration and open new therapeutic avenues.

### DJ-1 Is Required for Survival of the GIRK2-Positive SN Neurons

Why is there a specific requirement for Ret and DJ-1 activity in the GIRK2-positive subpopulation of SN neurons, considering that Ret and DJ-1 are expressed by most if not all DA neurons in SN and VTA [10],[27],[43]? GIRK2-positive neurons appear to be more sensitive than calbindin-positive neurons to toxic insult [44],[45] and calbindin-positive SN neurons are specifically spared in PD [46]. The presence and activity of GIRK2 itself may be a cause of vulnerability, since elevating the levels of GIRK2 further sensitizes these neurons [44]. Remarkably, GDNF was found to acutely modulate the excitability of midbrain dopaminergic neurons by inhibiting A-type K+ channels, a function that specifically involves the Mapk pathway [47]. Although the effects of Ret signaling on GIRK2 have not been studied, it is tempting to speculate that the modulation of Ras/Mapk signaling by Ret and DJ-1 also affects GIRK2 function and vulnerability of dopaminergic neurons. Further studies focusing specifically on the GIRK2 subpopulation will better define the exact biochemical processes involved in their survival and their interplay with other age-related cellular changes.

### DJ-1 Interacts Genetically with Constitutively Active Ret in *Drosophila*

Our results show that DJ-1 promotes survival of dopaminergic neurons only in conditions of aging and trophic insufficiency, suggesting that the function(s) of DJ-1 might only be uncovered in specific circumstances. The lack of a strong phenotype in DJ-1 null mutants has prevented the analysis of DJ-1 function in vivo. In vitro studies have suggested several functions for DJ-1 [18],[19],[26],[48],[49]; however, these proposed functions remain to be validated in vivo. Because fly genetics has previously uncovered PD-associated mechanisms [50], we chose to investigate genetic interactions between Ret and DJ-1 in the *Drosophila* eye. Constitutively active versions of Ret mediate excessive cell proliferation, abnormal increases in cell size, differentiation, and polarity defects. These defects induce a late-onset pro-apoptotic wave (in late pupal stages), resulting in adult eyes of reduced size with fewer ommatidia. Thus, even though Ret is a pro-survival regulator in mammalian systems, its excessive activation in the fly developing retina induces developmental abnormalities that indirectly lead to partial eye degeneration. Loss of endogenous *Drosophila* DJ-1A/B reduces the signaling output of activated Ret and largely alleviates these developmental defects. Photoreceptor cell size and the balance between photoreceptor proliferation and differentiation are returned to normal in a DJ-1A/B loss-of-function background. When we instead activated the PI3K/Akt pathway in the eye, endogenous DJ-1A/B were not required, nor was DJ-1A/B overexpression sufficient, to modulate this phenotype, indicating that DJ-1A/B do not interact with the PI3K/Akt pathway. This is in contrast to previous work that proposed DJ-1 to be a potent modulator of the PI3K/Akt pathway [19]. However, recent work by the same group suggests that DJ-1 may do so only in oncogenic (hypoxia) situations [36], and our results suggest that the interaction between DJ-1 and PTEN is PI3K/Akt-independent. Indeed, emerging evidence suggests lipid phosphatase-independent roles of PTEN [51], the best studied being a protein-phosphatase-dependent inhibition of Ras/MAPK signaling [52],[53], modulation of JNK signaling [54], and several nuclear functions, including control of cell cycle progression and maintenance of genomic stability [55].

### DJ-1 Interacts with Ras/ERK Signaling to Control *Drosophila* Eye and Wing Development

We found that DJ-1 is necessary and sufficient to mediate the effects of activated Ras during the development of the eye. Because DJ-1 failed to modulate constitutively active ERK/Mapk signalling during eye development, we propose that DJ-1 acts in parallel to Ras and either upstream or in parallel to ERK. We then investigated whether endogenous DJ-1B has any physiological function during the development of the fly. We found that DJ-1B is required, as an ERK/rolled interactor to control the development of photoreceptor neurons and wing venation. These observations establish a novel physiological role for DJ-1B in the intact fly. In vitro experiments previously suggested that DJ-1 might interact with Ras signaling. DJ-1 was first defined as a Ras-pathway interactor during oncogenic transformation [18] and a recent study reported that DJ-1 regulates the activation status of the ERK kinase in vitro [38].

### How Do Ret and DJ-1 Molecularly Interact in Aging SN Neurons?

Mechanistic studies in the Ret/DJ-1 mouse model are difficult to pursue, because of the region-specific and late-onset phenotype. Studies in which constitutively active or dominant negative Akt was virally delivered into the mouse brain suggested that Akt regulates the survival, cell size, and target innervation of SN neurons [56],[57]. Ret is a potent activator of both PI3K/Akt and Ras/ERK, and the loss of cell bodies, axons, and reduced cell size in *DAT-Ret* mice suggest possible defects in PI3K/Akt and/or Ras/ERK signaling. Our finding that DJ-1 does not interact with Akt signaling in *Drosophila* suggests that Akt signaling might not be the major pathway that cooperates with DJ-1 to regulate SN survival. Mice over-expressing activated Ras (Ras^V12^) in the nervous system have larger neurons and embryonic mesencephalic neurons derived from these mice are resistant to toxin*-*induced degeneration, suggesting that Ras signaling promotes survival of SN neurons [58]. A recent analysis of Ret knockin mice revealed a critical role for Ras/B-Raf/IKK signaling, but not for PI3K and ERKs, in the survival of sympathetic neurons [59]. It is therefore possible that DJ-1 cooperates with Ras-associated signaling to promote survival of aging SN neurons deprived of trophic support. It is also possible that DJ-1 and associated Ras signaling cooperate with PI3K/Akt signaling to control common downstream effectors and further studies will address this possibility.

### Aging, Trophic Insufficiency, and Cellular Stress Promote Dopaminergic Neurodegeneration

Deletion of both Ret and DJ-1 leads to a presymptomatic parkinsonian state in aging mice characterized by the lack of alpha-synuclein deposits, behavioural alterations, and loss of total dopamine, raising the possibility that the mechanisms regulating cell survival and target innervations might differ from those regulating protein homeostasis and dopamine dynamics. Compensatory mechanisms are likely to exist in the nigrostriatal system that maintains dopaminergic homeostasis below a certain threshold of SN neurodegeneration [1]. Several potential compensatory mechanisms have been previously described [60] and Ret/DJ-1 mice could serve as basis for further investigations of these mechanisms. The low penetrance of PD and the variability of symptoms in family members who inherit PD-associated mutations have raised the possibility that several risk factors interact to promote SN neuronal demise (the multiple hit hypothesis of PD [6]). A combination of high cytoplasmic calcium, elevated levels of free cytoplasmic dopamine, and the presence of alpha-synuclein induces selective death of cultured DA neurons, and interference with any of these individual hits alleviated neuronal cell death [61]. We report here a chronic genetic mouse model in which the interplay between three factors (aging, trophic insufficiency, and increased cellular stress due to DJ-1 inactivation) synergize and cause the loss of approximately 50% of GIRK2 DA neurons in the SN. Although the relevance of this three-component network to PD remains to be demonstrated, our findings underscore the importance of higher-order interactions between “sub-lethal” dopaminergic insults in promoting cell death.

In summary, we propose that the tight integration between aging, trophic signaling pathways, and the signaling network defined by PD-associated genes, including DJ-1, is critical for DA neuron survival. Further research on aging mechanisms coupled to studies on trophic factors and oxidative stress regulation may identify common denominators of these three processes and uncover new cellular targets for drug development in PD.

## Materials and Methods

### Animals

The generation of *Ret^lx^* [62] and *Dat-Cre* [63] alleles were described previously. The *DJ-1* ^−*/*−^ mice are described in a separate paper [25]. The GMR-dRet^MEN2^ and UAS-dRet^MEN2^ flies were generously provided by Ross Cagan (Mount Sinai). DJ-1A and DJ-1B knockout as well as UAS-DJ1A and UAS-DJ1B flies were a kind gift from Nancy Bonini (University of Pennsylvania). Wild-type, dominant negative (D954A), and constitutive active (CAAX) UAS-PI3K flies were from Sally Leevers (Cancer Research UK). Sev-Ras^G12V^ flies were from Marc Therrien (University of Montreal), and UAS-rl^SEM^ flies were kindly provided by Jongkyeong Chung (KAIST, Korea). UAS-dPTEN flies were a kind gift from Tak Mak (University of Toronto). All other fly lines were from the Bloomington Stock Center.

### Mouse Histology and Immunohistochemistry

Immunohistochemistry, stereology, and fiber density measurements were essentially performed as previously described [10]. Thirty µm-thick free floating sections were used for immunostainings. Primary antibodies were directed against: Tyrosine hydroxylase-TH (mouse monoclonal, 1∶2000, DiaSorin, Stillwater Massachusetts, USA), Pitx3 (rabbit polyclonal, generously provided by M.P. Smidt (Utrecht University), 1∶1000, [64]), GIRK2 (rabbit polyclonal, 1∶80, Alomone labs), Calbindin (mouse monoclonal, 1∶500, Sigma), Iba1 (1∶1000, rabbit polyclonal, Wako, Neuss, Germany), and GFAP (1∶500 rabbit polyclonal, DakoCytomation, Glostrup, Denmark). For immunofluorescence, sections were first premounted, and then the following primary antibodies were used: anti-TH antibody (mouse monoclonal, 1∶2000, DiaSorin, Stillwater Massachusetts, USA) or with anti-DAT (rat polyclonal, 1∶500, Chemicon/Millipore). For stereology, every sixth section spanning the ventral midbrain was used for measurements. To quantify the density of astrocytes in the dorsal striatum, one picture was acquired from every sixth section of the dorsal striatum. Six to eight sections were analyzed/animal and at least 4 animals were analyzed per group.

### Quantification of Soma Size for SN Neurons

GIRK2 immunostained coronal sections were analyzed using a bright field microscope with a 40× objective. Random cells were selected with stereological methods using the StereoInvestigator software. Five to seven animals per group were analyzed by circling cell soma of 149–275 cells per animal.

### Measurements of Dopamine Levels

Eighteen-mo-old mice were sacrificed, brains were removed, snap-frozen, and the striata were dissected. The tissue was homogenized in 0.1 M perchloric acid containing 0.5 mM disodium EDTA and 50 ng/ml, 3,4-dihydroxybenzylamine as an internal standard, centrifuged at 50,000 g for 30 min, and filtered through a 0.22 µM PVDF membrane. The samples were subjected to HPLC analysis as described previously [10].

### Open Field Analysis

To test general activity of aging control and mutant mice, mice were subjected to open field behavioral assessment. Eighteen-mo-old mice were housed individually in a room with 12 h/12 h reversed day-night cycle. All experiments were conducted during the night period in a quiet room by 12 lux light. Mice were placed into a 59 cm×59 cm large arena for 20 min, and their movement was followed using EthoVision Pro 2.2. (Noldus, Sterling, USA). The experiment was repeated on the consecutive day and the average distance each mouse travelled during the two trials was determined. Experimental protocols were approved by the government of Oberbayern, Germany.

### Fly Imaging and Toluidine Blue Stainings

Pictures of P1–P5 eyes and wings were acquired using a Leica MZ 9.5 stereomicroscope equipped with a Leica DFC320 digital camera (LeicaMicrosystems, Wetzlar, Germany). For toluidine blue stainings, heads from P1–P5 animals were dissected and post-fixed in 2.5% glutaraldehyde. After washing with PBS, heads were incubated in a 1% osmium tertaoxide solution (Science Services, Munich, Germany), then dehydrated in ethanol solutions of increasing concentrations, followed by a 10 min incubation in propylene oxide. Heads were then incubated overnight in a solution containing 50% propylene oxide and 50% durcupan epoxy resin, which contained 48% component A/M, 40% hardener B, 2.25% accelerator C, and 9% plasticizer D (Sigma-Aldrich). Then, heads were incubated overnight in 100% durcupan epoxy resin. The next day, heads and fresh durcupan resin were transferred to molds, oriented tangentially, and then cooked overnight at 60°C. The heads were then removed from molds and cut using a 2088 ultrotome (LKB, Bromma, Sweden). Three µm-thick sections were collected, mounted, and then stained using a pre-warmed toluidine blue solution that contained 0.1% toluidine blue (Serva Electrophoresis, Heidelberg, Germany) and 2.5% sodium carbonate. After a quick wash in water, sections were allowed to dry and were then covered with paraffin oil. Pictures at different retinal depths were acquired for each head. To determine ommatidium size and the number of photoreceptor neurons/ommatidium, at least 150 ommatidia/animal from at least 4 animals were analyzed.

### RT-PCR Analysis

Heads and bodies were separated from 10 WT and DJ-1^−/−^ flies and snap frozen in liquid nitrogen. RNA preparation was performed using the RNAeasy kit and the QIAshredder spin column (Qiagen) according to the manufacturer's instructions. The RNA concentration was determined using a NanoDrop ND1000 spectrometer, and 10 ng per sample of total RNA was subjected to RT-PCR amplification with 25, 30, or 35 cycles using the Qiagen OneStep RT-PCR kit according to the manufacturer's instructions. The following exon-spanning primer pairs were used: 5′-CAAGCAAGCCGATAGATAAACA-3′ (GAPDH forward) 5′-CAAGTGAGTGGATGCCTTGT-3′ (GAPDH reverse) 5′-GGAAAGATCCTTGTTACCGTG-3′(DJ-1A forward) 5′-CCATCCTGGACCACAGTCTT-3′ (DJ-1A reverse).

### DNA Constructs and RNA Interference

A pCMV-myc-DJ-1 construct and the empty pCMV-Myc vector were acquired as a kind gift from Phillip Kahle (Hertie Institute, Tübingen, Germany). SiRNA oligonucleotides (stealth-siRNA, Invitrogen) had the following sequences: (DJ-1) AGGAAAUGGAGACGGUCAU-CCCUGU; (CTRL) ACAGGGAUGACCGUCUCCAUUUCCU. The sequences have been described previously and validated for off target effects [13],[48].

### Cell Culture and Transfections

MEFs were isolated from E13.5 WT or DJ-1^−/−^ embryos according to standard procedures. Experiments were performed at passage 4–6. MEFs, HeLa cells, and A549 cells were cultured in DMEM supplemented with 10% serum, 1% L-Glutamine, and 1% pen/strep. SH-SY5Y cells (ATCC #CRL-2266) were cultured in the DMEM/F12 (1∶1) supplemented with 10% serum, GlutaMAX, and 1% pen/strep. MEFs were transfected using the standard CaPO_4_-precipitate method. HeLa cells were transfected using Lipofectamine 2000 (Invitrogen) for plasmids or Lipofectamine RNAiMAX (Invitrogen) for siRNA by the forward transfection method, according to manufacturer's instructions. SH-SY5Y and A549 cells were transfected using Lipofectamine 2000 (Invitrogen) for plasmids or Lipofectamine RNAiMAX (Invitrogen) by the reverse transfection method, according to the manufacturer's instructions. Plasmid overexpression experiments were incubated for 24 h after transfections before analysis for all cell types. SiRNA knockdowns were incubated for 48 h after transfection for HeLa and A549 cells, while SH-SY5Y cells were incubated for 96 h after transfection.

### Immunoblotting

Fly P1–P5 heads from at least 50 animals were collected and snap frozen in liquid nitrogen, then stored at −80°C. The lysis and detection were performed as previously described [15] using an anti-DJ1A/B antibody (rabbit polyclonal, 1∶500, kind gift from Leo Pallanck). Cultured cell lines were harvested in a lysis buffer containing 1% Triton X-100, 150 mM NaCl, 1 mM EDTA, 10 mM Tris-HCl (pH 7.5), 100 mM NaF, 1 mM NaVO_3_, 10 mM Na_4_P_2_O_7_, and Complete protease inhibitor mixture (Roche Diagnostics). Mouse brains were snap frozen, ventral midbrains and striata were dissected on ice, and homogenized in a buffer containing 150 mM NaCl, 50 mM Tris_HCl, pH 7.4, 2 mM EDTA, 1% Nonidet P-40, 1% SDS, and Complete protease inhibitor mixture (Roche Diagnostics) by 10 strokes in a dounce homogenizer. Cell lysates and brain homogenates were centrifuged at 1,000 g for 10 min, supernatants were saved, and the protein concentration was determined using the D_C_ protein assay (BioRad). Samples were subjected to SDS-PAGE and Immunoblotting according to standard techniques. The following antibodies were used: anti-AKT (9272, Cell Signaling Technology), anti-phospho-AKT (Ser473, 9271, Cell Signaling Technology), DJ-1 (ab4150, Abcam), anti-phospho-p42/p44 MAPK (4376, Cell Signaling Technology), anti-p42/p44 MAPK (9102, Cell Signaling Technology), anti-Ret (70R-RG002, Fitzgerald), and anti-β-tubulin (T-8660, Sigma).

## Supporting Information

Figure S1 **Ret and DJ-1 expression in the nigrostriatal system of the mouse and in SH-SY5Y cells.** (A,B) Immunoblots from mouse ventral midbrain extracts (18 mo) or striatum (24 mo) of control, *DJ-1* ^−*/*−^, *DAT-Cre;+/+ (Cre)*, and *Dat-Ret* mice, incubated with DJ-1, ERK1/2, Ret, and β-actin antibodies, as indicated. (B,C) Quantification of blot A. Average levels of DJ-1 protein (B) and Ret protein (C) in control, *DJ-1* ^−*/*−^, and *Dat-Ret* mice (*n* = 3 mice each) normalized against ERK1/2 levels. n.s., not significant. (E,F) Quantification of blot D. Average levels of DJ-1 protein (E) and Ret protein (F) in control, *DJ-1* ^−*/*−^, and *Dat-Ret* mice (*n* = 3 mice each) normalized against β-actin levels. n.s., not significant. (G) SH-SY5Y cells were treated with DJ-1 or CTRL siRNA and harvested after 96 h. No changes in endogenous Ret protein levels were observed after DJ-1 knockdown; α-tubulin was used as loading control. (H) SH-SY5Y cells were starved in 0.5% serum for 96 h, followed by treatment with 10% serum or a mixture of GDNF/GFRα1 (50 ng/ml) for 12 h. No changes in DJ-1 protein levels were observed after treatment; α-tubulin was used as loading control.
(7.73 MB TIF)

Figure S2 **Genetic interaction between Ret signaling and DJ-1 modulates development of the** ***Drosophila*** **eye.** (A–F) Images of the normal eye of an adult control fly (A), the smaller rougher eye of a fly overexpressing dRet^MEN2B^ controlled by the GMR promoter (B), and rescued eyes of *GMR-dRet^MEN2B^* flies carrying either deficiencies removing the *DJ-1A* (C) or *DJ-1B* (E) genes or insertions in the *DJ-1A* (D) or *DJ-1B* (F) genes. (G) Quantification of eye sizes in the indicated mutant and control flies (*n*>15 eyes per genotype; *** *p*<0.001, *t* test). (H–J) Photomicrographs of ultrathin eye sections stained with toluidine blue showing the normal size and pattern of individual ommatidia of an adult control fly (H), the larger ommatidia of a *GMR-dRet^MEN2B^* fly (I), and the near normal ommatidia of a *GMR- dRet^MEN2B^;Df(2R)CX1* fly (J). (K) Quantification of ommatidia sizes in the indicated mutant and control flies (*n* = 4 eyes per genotype; *** *p*<0.001, *t* test). (L–N) Overexpression of dRet^MEN2A^ controlled by the GMR promoter (*GMR-dRet^MEN2A^*) leads to a strong eye phenotype (M) compared to a control fly (L), which can be partially rescued by reducing DJ-1A/B levels (N). In *GMR-dRet^MEN2A^; DJ-1A^+/^* ^−^ *DJ-1B* ^−*/*−^ flies, eye size was restored (N). (O) Quantification of eye sizes in the indicated mutant and control flies (*n*>10 eyes per genotype; *** *p*<0.001, *t* test).
(7.89 MB TIF)

Figure S3 **Study of interactions between DJ-1A/B and PI3K and PTEN.** (A–E) Images of the normal eye of an adult control fly (A), the bigger eye of a fly overexpressing constitutively active PI3K (PI3K^CAAX^) controlled by GMR promoter (B), and eyes in which PI3KCAAX was co-overexpressed with DJ-1A (C), DJ-1B (D), or expressed in a DJ-1B heterozygous knockout background (E). Manipulation of DJ-1A/B levels failed to modulate the effects of PI3K overexpression (compare B to C–E). (F–J) Images of the normal eye of an adult control fly (F), the smaller and rough eye of a fly overexpressing PTEN controlled by the eyeless promoter (G), and rescued eyes of *eyeless-PTEN* flies that co-overexpress Ret^WT^ (H), DJ-1A (I), or DJ-1B (J). (K–O) Photomicrographs of ultrathin eye sections stained with toluidine blue showing the normal size and pattern of individual ommatidia of an adult control fly (K), the smaller ommatidia of an *eyeless-PTEN* fly (L), and the rescue of ommatidial sizes in *eyeless-PTEN* flies that co-overexpress RetWT (M), DJ-1A (N), or DJ-1B (O). (U) Quantification of eye sizes in the indicated mutant and control flies (*n*>15 eyes per genotype; *** *p*<0.001, *t* test). (V) Quantification of eye sizes in the indicated mutant and control flies (*n*>25 eyes per genotype; * *p*<0.05 and *** *p*<0.001, *t* test). (X) Quantification of ommatidia sizes in the indicated mutant and control flies (*n* = 4 eyes per genotype; * *p*<0.05 and ** *p*<0.01, *t* test). (P–T) Little effect of DJ-1B inactivation on the phenotype induced by PTEN overexpression. Images of the normal eye of an adult control fly (P), the slightly reduced eye of a fly overexpressing PTEN controlled by the GMR promoter (Q), and eye of similar size in GMR-PTEN flies heterozygous knockout for DJ-1A (E) or DJ-1B (S) or homozygous knockout for DJ-1B (T).
(7.64 MB TIF)

Figure S4 **DJ-1 does not regulate Akt or Erk1/2 phosphorylation in mammalian cell culture.** (A–D) Immunoblots from total cells lysates, incubated with phospho-Akt (S473), total Akt, phospho-Erk1/2, total Erk1/2, and DJ-1 antibodies, as indicated. (A) *WT* (+/+) and *DJ-1* ^−*/*−^ (−/−)MEFs were serum-starved, and treated with Insulin-like growth factor-1 (IGF-1) for 1, 15, or 120 min. Akt phosphorylation and Erk1/2 phosphorylation peaked at 15 min in both cases; no difference between *WT* and *DJ-1* ^−*/*−^ cells was observed. (B) *DJ-1* ^−*/*−^ MEFs were transiently transfected with a GFP control plasmid or a myc-DJ-1 plasmid; no differences in basal levels of phospho-Akt or phospho-Erk1/2 were observed. (C–D) HeLa (C), SH-SY5Y (C,D), and A549 (C) cell lines were transiently transfected with DJ-1 siRNA or a mutated CTRL siRNA (C,D). HeLa cells (C) or SH-SY5Y cells (D) were transiently transfected with a DJ-1-myc plasmid or the empty vector. No differences in phospho-Akt (C) or phospho-Erk1/2 (D) were observed.
(7.95 MB TIF)

## Abbreviations

DAT: dopamine transporter
dRet: *Drosophila* Ret
GDNF: glial cell line-derived neurotrophic factor
GFAP: glial fibrillary acidic protein
MEFs: mouse embryonic fibroblasts
PD: Parkinson disease
PI: phosphatidylinositol
RTK: receptor tyrosine kinase
SN: substantia nigra
TH: tyrosine hydroxylase
VTA: ventral tegmental area
