# Supplementary figures and images for: Pro-Survival Role for Parkinson's Associated Gene DJ-1 Revealed in Trophically Impaired Dopaminergic Neurons
Source: PLoS Biol. 2010 Apr 6;8(4):e1000349. doi: 10.1371/journal.pbio.1000349 (PMC2850379; doi:10.1371/journal.pbio.1000349)

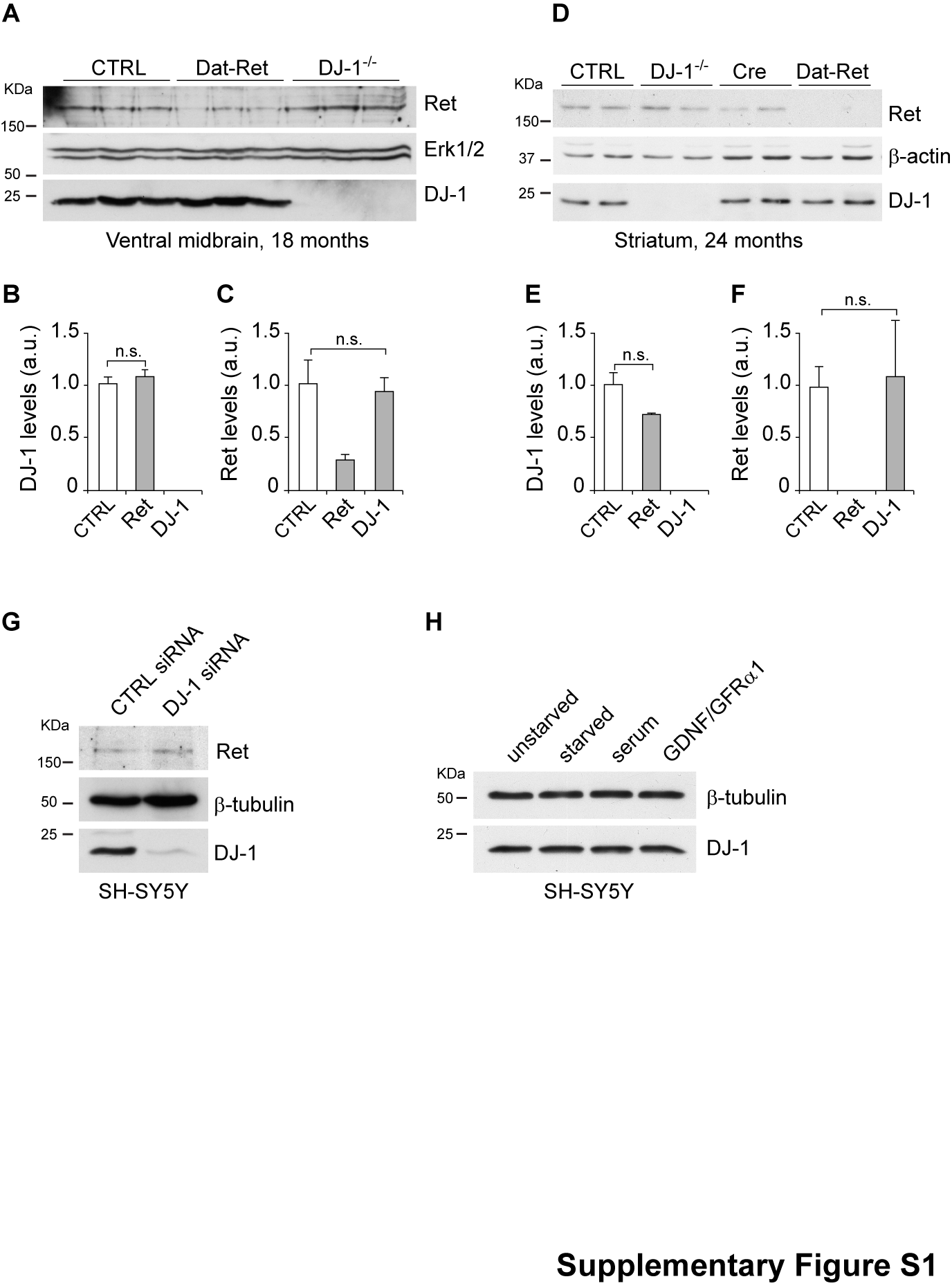

Supplement: Figure S1 — Ret and DJ-1 expression in the nigrostriatal system of the mouse and in SH-SY5Y cells. (A,B) Immunoblots from mouse ventral midbrain extracts (18 mo) or striatum (24 mo) of control, DJ-1 −/−, DAT-Cre;+/+ (Cre), and Dat-Ret mice, incubated with DJ-1, ERK1/2, Ret, and β-actin antibodies, as indicated. (B,C) Quantification of blot A. Average levels of DJ-1 protein (B) and Ret protein (C) in control, DJ-1 −/−, and Dat-Ret mice (n = 3 mice each) normalized against ERK1/2 levels. n.s., not significant. (E,F) Quantification of blot D. Average levels of DJ-1 protein (E) and Ret protein (F) in control, DJ-1 −/−, and Dat-Ret mice (n = 3 mice each) normalized against β-actin levels. n.s., not significant. (G) SH-SY5Y cells were treated with DJ-1 or CTRL siRNA and harvested after 96 h. No changes in endogenous Ret protein levels were observed after DJ-1 knockdown; α-tubulin was used as loading control. (H) SH-SY5Y cells were starved in 0.5% serum for 96 h, followed by treatment with 10% serum or a mixture of GDNF/GFRα1 (50 ng/ml) for 12 h. No changes in DJ-1 protein levels were observed after treatment; α-tubulin was used as loading control. (7.73 MB TIF) [file pbio.1000349.s001.tif]

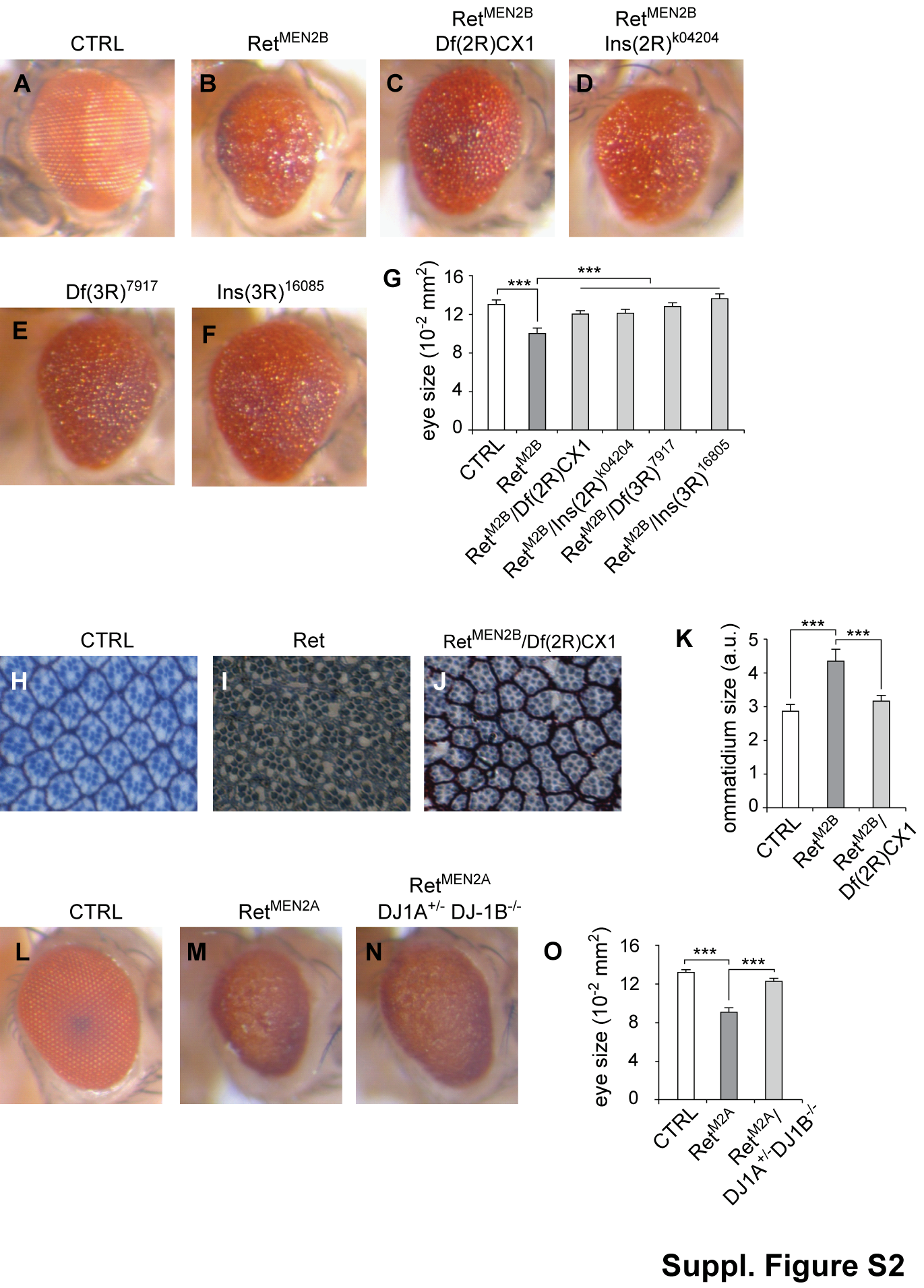

Supplement: Figure S2 — Genetic interaction between Ret signaling and DJ-1 modulates development of the Drosophila eye. (A–F) Images of the normal eye of an adult control fly (A), the smaller rougher eye of a fly overexpressing dRetMEN2B controlled by the GMR promoter (B), and rescued eyes of GMR-dRetMEN2B flies carrying either deficiencies removing the DJ-1A (C) or DJ-1B (E) genes or insertions in the DJ-1A (D) or DJ-1B (F) genes. (G) Quantification of eye sizes in the indicated mutant and control flies (n>15 eyes per genotype; *** p<0.001, t test). (H–J) Photomicrographs of ultrathin eye sections stained with toluidine blue showing the normal size and pattern of individual ommatidia of an adult control fly (H), the larger ommatidia of a GMR-dRetMEN2B fly (I), and the near normal ommatidia of a GMR- dRetMEN2B;Df(2R)CX1 fly (J). (K) Quantification of ommatidia sizes in the indicated mutant and control flies (n = 4 eyes per genotype; *** p<0.001, t test). (L–N) Overexpression of dRetMEN2A controlled by the GMR promoter (GMR-dRetMEN2A) leads to a strong eye phenotype (M) compared to a control fly (L), which can be partially rescued by reducing DJ-1A/B levels (N). In GMR-dRetMEN2A; DJ-1A+/ − DJ-1B −/− flies, eye size was restored (N). (O) Quantification of eye sizes in the indicated mutant and control flies (n>10 eyes per genotype; *** p<0.001, t test). (7.89 MB TIF) [file pbio.1000349.s002.tif]

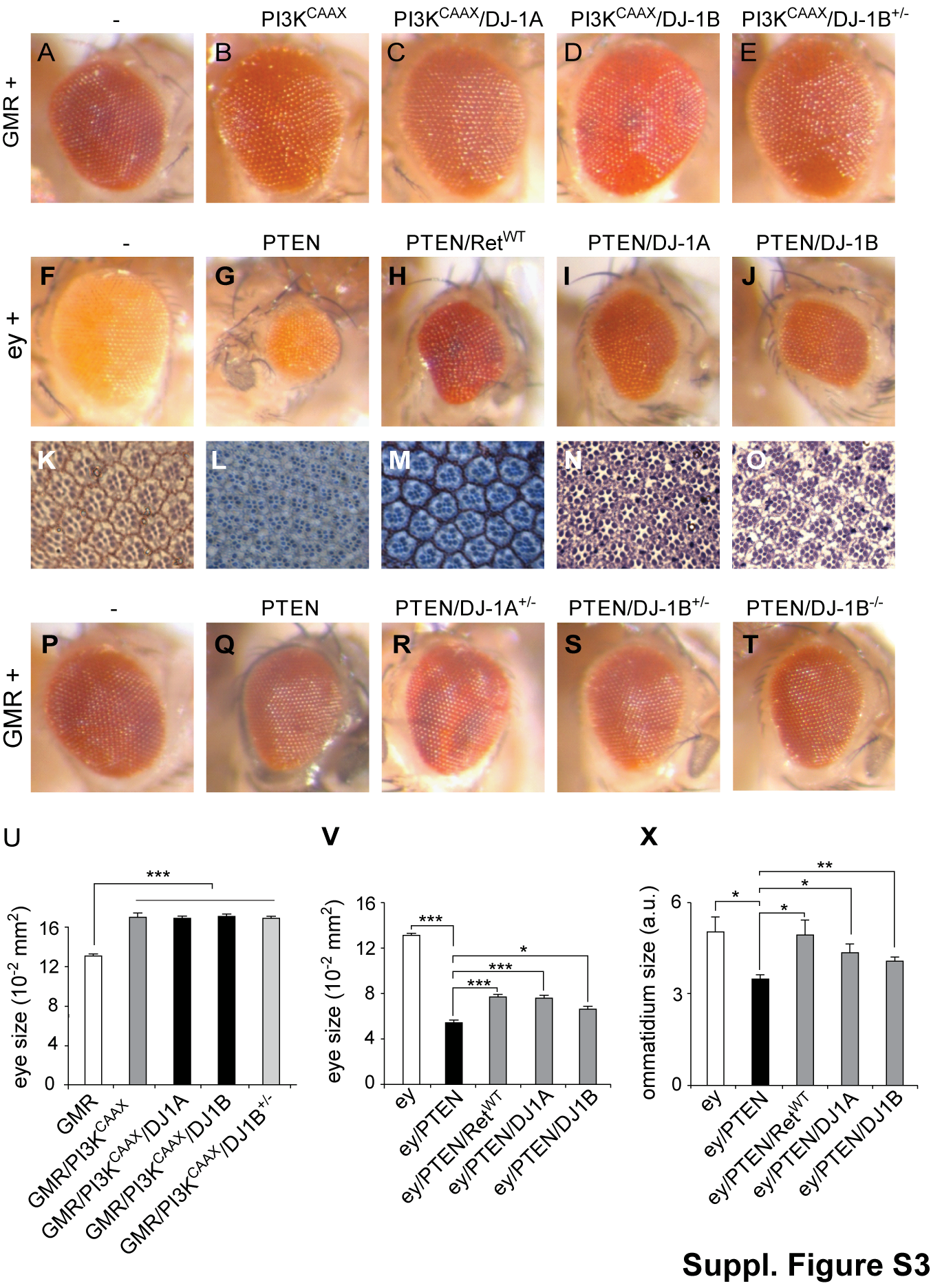

Supplement: Figure S3 — Study of interactions between DJ-1A/B and PI3K and PTEN. (A–E) Images of the normal eye of an adult control fly (A), the bigger eye of a fly overexpressing constitutively active PI3K (PI3KCAAX) controlled by GMR promoter (B), and eyes in which PI3KCAAX was co-overexpressed with DJ-1A (C), DJ-1B (D), or expressed in a DJ-1B heterozygous knockout background (E). Manipulation of DJ-1A/B levels failed to modulate the effects of PI3K overexpression (compare B to C–E). (F–J) Images of the normal eye of an adult control fly (F), the smaller and rough eye of a fly overexpressing PTEN controlled by the eyeless promoter (G), and rescued eyes of eyeless-PTEN flies that co-overexpress RetWT (H), DJ-1A (I), or DJ-1B (J). (K–O) Photomicrographs of ultrathin eye sections stained with toluidine blue showing the normal size and pattern of individual ommatidia of an adult control fly (K), the smaller ommatidia of an eyeless-PTEN fly (L), and the rescue of ommatidial sizes in eyeless-PTEN flies that co-overexpress RetWT (M), DJ-1A (N), or DJ-1B (O). (U) Quantification of eye sizes in the indicated mutant and control flies (n>15 eyes per genotype; *** p<0.001, t test). (V) Quantification of eye sizes in the indicated mutant and control flies (n>25 eyes per genotype; * p<0.05 and *** p<0.001, t test). (X) Quantification of ommatidia sizes in the indicated mutant and control flies (n = 4 eyes per genotype; * p<0.05 and ** p<0.01, t test). (P–T) Little effect of DJ-1B inactivation on the phenotype induced by PTEN overexpression. Images of the normal eye of an adult control fly (P), the slightly reduced eye of a fly overexpressing PTEN controlled by the GMR promoter (Q), and eye of similar size in GMR-PTEN flies heterozygous knockout for DJ-1A (E) or DJ-1B (S) or homozygous knockout for DJ-1B (T). (7.64 MB TIF) [file pbio.1000349.s003.tif]

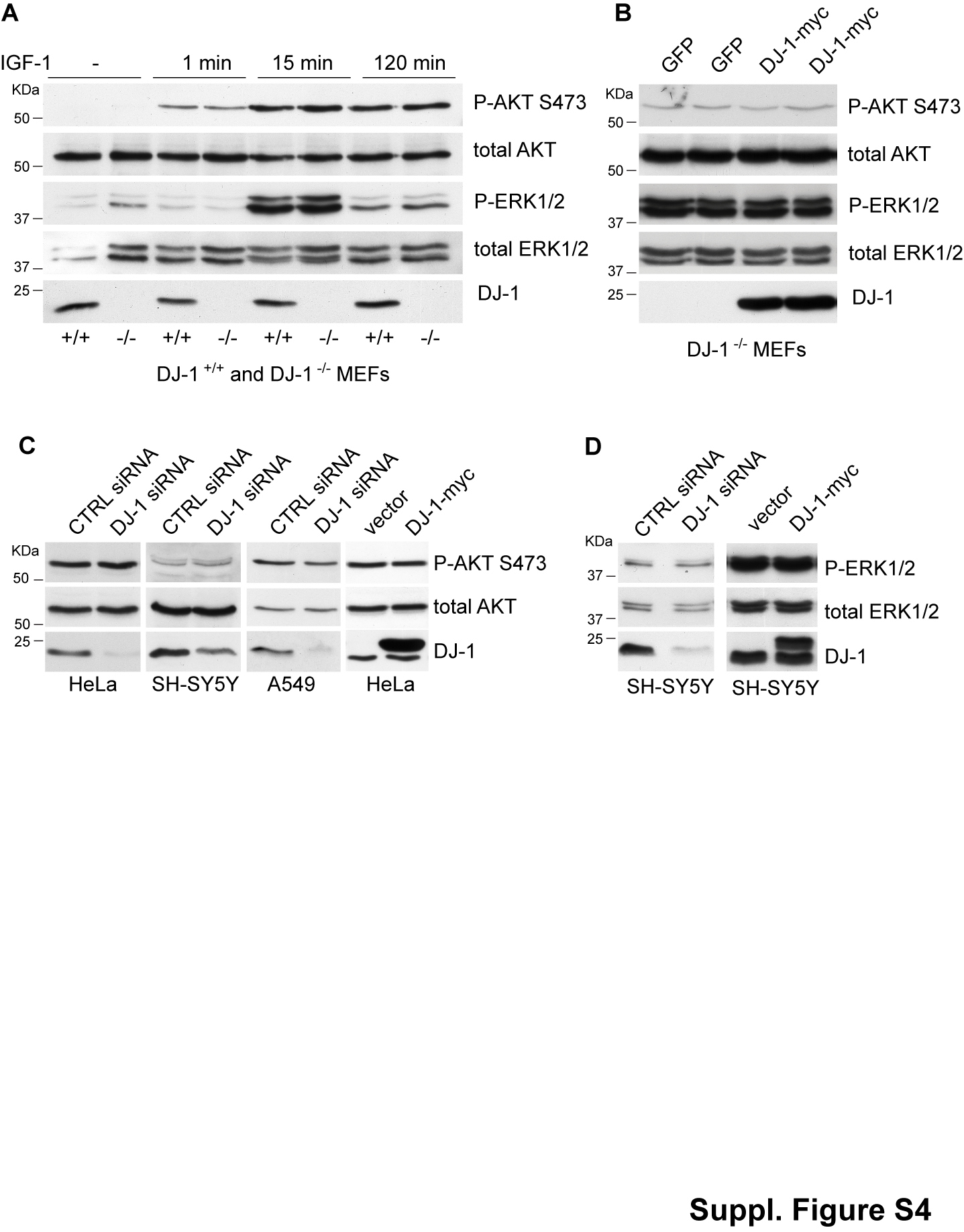

Supplement: Figure S4 — DJ-1 does not regulate Akt or Erk1/2 phosphorylation in mammalian cell culture. (A–D) Immunoblots from total cells lysates, incubated with phospho-Akt (S473), total Akt, phospho-Erk1/2, total Erk1/2, and DJ-1 antibodies, as indicated. (A) WT (+/+) and DJ-1 −/− (−/−)MEFs were serum-starved, and treated with Insulin-like growth factor-1 (IGF-1) for 1, 15, or 120 min. Akt phosphorylation and Erk1/2 phosphorylation peaked at 15 min in both cases; no difference between WT and DJ-1 −/− cells was observed. (B) DJ-1 −/− MEFs were transiently transfected with a GFP control plasmid or a myc-DJ-1 plasmid; no differences in basal levels of phospho-Akt or phospho-Erk1/2 were observed. (C–D) HeLa (C), SH-SY5Y (C,D), and A549 (C) cell lines were transiently transfected with DJ-1 siRNA or a mutated CTRL siRNA (C,D). HeLa cells (C) or SH-SY5Y cells (D) were transiently transfected with a DJ-1-myc plasmid or the empty vector. No differences in phospho-Akt (C) or phospho-Erk1/2 (D) were observed. (7.95 MB TIF) [file pbio.1000349.s004.tif]
